# Benchmarking health system performance across regions in Uganda: a systematic analysis of levels and trends in key maternal and child health interventions, 1990–2011

**DOI:** 10.1186/s12916-015-0518-x

**Published:** 2015-12-03

**Authors:** D. Allen Roberts, Marie Ng, Gloria Ikilezi, Anne Gasasira, Laura Dwyer-Lindgren, Nancy Fullman, Talemwa Nalugwa, Moses Kamya, Emmanuela Gakidou

**Affiliations:** Institute for Health Metrics and Evaluation, University of Washington, 2301 5th Ave, Suite 60, Seattle, WA 98121 USA; Infectious Diseases Research Collaboration, Mulago Hospital Complex, Kampala, Uganda; African Leaders Malaria Alliance, Kampala, Uganda; School of Medicine, Makerere University College of Health Sciences, Kampala, Uganda

**Keywords:** Coverage, Indicators, Inequalities, Maternal and child health, Subnational benchmarking, Uganda, Under-5 mortality

## Abstract

**Background:**

Globally, countries are increasingly prioritizing the reduction of health inequalities and provision of universal health coverage. While national benchmarking has become more common, such work at subnational levels is rare. The timely and rigorous measurement of local levels and trends in key health interventions and outcomes is vital to identifying areas of progress and detecting early signs of stalled or declining health system performance. Previous studies have yet to provide a comprehensive assessment of Uganda’s maternal and child health (MCH) landscape at the subnational level.

**Methods:**

By triangulating a number of different data sources – population censuses, household surveys, and administrative data – we generated regional estimates of 27 key MCH outcomes, interventions, and socioeconomic indicators from 1990 to 2011. After calculating source-specific estimates of intervention coverage, we used a two-step statistical model involving a mixed-effects linear model as an input to Gaussian process regression to produce regional-level trends. We also generated national-level estimates and constructed an indicator of overall intervention coverage based on the average of 11 high-priority interventions.

**Results:**

National estimates often veiled large differences in coverage levels and trends across Uganda’s regions. Under-5 mortality declined dramatically, from 163 deaths per 1,000 live births in 1990 to 85 deaths per 1,000 live births in 2011, but a large gap between Kampala and the rest of the country persisted. Uganda rapidly scaled up a subset of interventions across regions, including household ownership of insecticide-treated nets, receipt of artemisinin-based combination therapies among children under 5, and pentavalent immunization. Conversely, most regions saw minimal increases, if not actual declines, in the coverage of indicators that required multiple contacts with the health system, such as four or more antenatal care visits, three doses of oral polio vaccine, and two doses of intermittent preventive therapy during pregnancy. Some of the regions with the lowest levels of overall intervention coverage in 1990, such as North and West Nile, saw marked progress by 2011; nonetheless, sizeable disparities remained between Kampala and the rest of the country. Countrywide, overall coverage increased from 40 % in 1990 to 64 % in 2011, but coverage in 2011 ranged from 57 % to 70 % across regions.

**Conclusions:**

The MCH landscape in Uganda has, for the most part, improved between 1990 and 2011. Subnational benchmarking quantified the persistence of geographic health inequalities and identified regions in need of additional health systems strengthening. The tracking and analysis of subnational health trends should be conducted regularly to better guide policy decisions and strengthen responsiveness to local health needs.

**Electronic supplementary material:**

The online version of this article (doi:10.1186/s12916-015-0518-x) contains supplementary material, which is available to authorized users.

## Background

The measurement of population health outcomes and intervention coverage is a vital component of evaluating health systems performance [1]. The allocation of resources by policymakers should be guided by evidence of gaps in coverage and opportunities to improve health outcomes. While national-level health indicators are commonly used for benchmarking and target setting, subnational coverage can be much more informative, revealing geographic variance and allowing decision-makers to tailor policies to local conditions [2]. However, estimates of subnational levels and trends for health indicators are often unavailable in low-resource settings, largely due to data scarcity and insufficient health information systems [3].

Uganda has made marked progress in reducing under-5 mortality since 1990, which declined from 163 deaths per 1,000 live births in 1990 to 85 deaths per 1,000 live births in 2013 [4]. While Uganda has witnessed a slight increase in maternal mortality since 1990, the country has experienced an annualized rate of decline of 3.2 % since its peak maternal mortality ratio of 475 deaths per 100,000 live births in 2001 [5]. Despite these national-level trends, it is unknown whether the declines have been realized equally across subnational areas or to what extent geographic inequalities have changed over time. Subnational monitoring is critical for Uganda to maximize impact by targeting high-burden areas.

Over the last two decades, Uganda’s government has prioritized expanding health services across a number of key maternal and child health (MCH) interventions. Since 2002, distribution of insecticide-treated nets (ITNs) has substantially expanded [6], indoor residual spraying (IRS) has been implemented in select districts [7], and artemisinin-based combination therapies (ACTs) have been introduced as the first-line treatment for uncomplicated malaria since 2006 [8]. The pentavalent vaccine replaced the original diphtheria-pertussis-tetanus formulation in 2002 [9], and Gavi, the Vaccine Alliance, has provided health systems strengthening and immunization services support (ISS) periodically since 2001 [10]. The extent to which these efforts have resulted in improved intervention access and use throughout the country is unknown. Tracking local trends and generating sound subnational estimates of intervention coverage are vital components of evaluating the success of the implementation of policies and programs aiming to improve MCH services.

To date, few studies have benchmarked health systems performance at the subnational level in low-income countries. Several studies have addressed inequalities in intervention coverage but are generally restricted to comparisons by sex, urbanicity, or socioeconomic status [11–13]. Others have applied small-area techniques to estimating intervention coverage, but these studies have generally been limited to a subset of health indicators or to a restricted time range [14–16]. More recently, a study in Zambia assessed subnational health systems performance across a wide swathe of health indicators [17], but similarly comprehensive studies have not been conducted in Uganda. Previous work in Uganda includes the government’s annual district league tables, which rely on administrative records to rank districts [18]. However, reporting through the Health Management Information System (HMIS) is often incomplete and inaccurate, and reliable denominator estimates are difficult to obtain [19]. Other efforts using household surveys have been limited to using a single cross-sectional survey to estimate district-level child mortality in 2006 and underweight and obesity prevalence in 2011 [20, 21].

By systematically collating all available data, this study provides a comprehensive regional assessment of a broad range of MCH indicators and socioeconomic factors in Uganda from 1990 to 2011.

## Methods

### Data and indicator selection

We identified data sources for MCH indicators via in-country meetings with collaborators and relevant stakeholders. For this analysis, we used 17 household surveys and two population censuses (Table 1). While we identified administrative data sources including drug distribution from National Medical Stores, ITN distribution campaigns, and HMIS data, we excluded them from our analysis due to concerns about completeness and lack of reliable denominator information.

**Table 1:** Definition of indicators

Indicator	Definition	Data sources
Health outcomes		
Under-5 mortality	The probability that a child born in the given year will die before reaching the age of 5 years, expressed in terms of under-5 deaths per 1,000 live births	DHS: 1995, 2000–2001, 2006, 2011
		AIS: 2011
		Census: 1991, 2002
Percentage of children under 5 who are underweight	The proportion of children aged 6–59 months who are underweight, as defined by weighing two or more standard deviations below the international anthropometric reference population median of weight for age	DHS: 1995, 2000–2001, 2006, 2011
		IHS: 1992–1993
		UNHS: 1999–2000
		UNPS: 2009–2010, 2010–2011
Prevalence of stunting among children under 5	The proportion of children aged 6–59 months who are stunted, as defined by weighing two or more standard deviations below the international anthropometric reference population median of height for age	DHS: 1995, 2000–2001, 2006, 2011
		IHS: 1992–1993
		UNHS: 1999–2000
		UNPS: 2009–2010, 2010–2011
Malaria interventions		
Insecticide-treated net (ITN) ownership	The proportion of households that owned at least one ITN	DHS: 2000–2001, 2006, 2011
		MIS: 2009–2010
		Netmark: 2000, 2006
ITN use by children under 5	The proportion of children under 5 who slept under an ITN during the night before the survey	DHS: 2000–2001, 2006, 2011
		MIS: 2009–2010
		Netmark: 2000, 2006
		UNHS: 2005–2006, 2009–2010
Indoor residual spraying (IRS)	The proportion of households that were sprayed with an insecticide-based solution in the last 12 months	DHS: 2006, 2011
		MIS: 2009–2010
ITN ownership or IRS	The proportion of households that owned at least one ITN or were sprayed with an insecticide-based solution in the last 12 months, or both	DHS: 2006, 2011
		MIS: 2009–2010
ITN use or IRS	The proportion of children under 5 who slept under an ITN during the night before the survey, or lived in a household that received IRS in the last 12 months, or both	DHS: 2006, 2011
		MIS: 2009–2010
Percentage of febrile children under 5 who received artemisinin-based combination therapies (ACTs)	The proportion of children under 5 with a fever in the last two weeks who received ACTs	DHS: 2006, 2011
		MIS: 2009–2010
Percentage of antimalarials provided that were ACTs	The proportion of malaria treatments that were ACTs and given to children under 5 with fever in the last two weeks	DHS: 2006, 2011
		MIS: 2009–2010
Childhood immunizations		
Bacillus Calmette-Guérin immunization (BCG, 0–12; BCG, u5)	The proportion of children (1) aged 0–12 months or (2) aged under 5 who were vaccinated against tuberculosis	DHS: 1995, 2000–2001, 2006, 2011
		IHS: 1992–1993
		UNHS: 1999–2000
		UNSDS: 2004, 2008
Measles immunization	The proportion of children aged 12–24 months who received measles vaccination	DHS: 1995, 2000–2001, 2006, 2011
		IHS: 1992–1993
		UNHS: 1999–2000
		UNPS: 2009–2010, 2010–2011
		UNSDS: 2004, 2008
Oral polio immunization, three doses (OPV3)	The proportion of children aged 12–59 months who received three doses of OPV	DHS: 1995, 2000–2001, 2006, 2011
		IHS: 1992–1993
		UNHS: 1999–2000
		UNSDS: 2004, 2008
Pentavalent immunization	The proportion of children aged 12–59 months who received three doses of the pentavalent vaccine, which includes protection against diphtheria-pertussis-tetanus, hepatitis B, and *Haemophilus influenzae* type b	DHS: 2006, 2011
		UNSDS: 2004, 2008
Other key MCH interventions		
Antenatal care, one and four visits (ANC1 and ANC4)	The proportion of women aged 15–49 who gave birth in the given year and had one/four or more antenatal visits attended by skilled personnel (doctor, nurse, midwife, or clinical officer) at a health facility during the corresponding pregnancy	DHS: 1995, 2000–2001, 2006, 2011
		MIS: 2009–2010
		AIS: 2011
		IHS: 1992–1993
		UNHS: 1999–2000
Percentage of women who received two or more doses of the tetanus toxoid vaccine during pregnancy	The proportion of women aged 15–49 who received two or more doses of the tetanus toxoid vaccine during their last pregnancy	DHS: 1995, 2000–2001, 2006, 2011
		UNHS: 1999–2000
Skilled birth attendance	The proportion of women 15–49 who gave birth in the given year and delivered with a skilled birth attendant (a doctor, nurse, midwife, or clinical officer)	DHS: 1995, 2000–2001, 2006, 2011
		UNHS: 1999–2000
Exclusive breastfeeding	The proportion of children aged 0–5 months who consumed only breast milk the night before the survey	DHS: 1995, 2000–2001, 2006, 2011
Receipt of oral rehydration salts in response to diarrhea (ORS)	The proportion of children under 5 who had diarrhea in the past two weeks and who received ORS	DHS: 1995, 2000–2001, 2006, 2011
Care at health facility after suspected acute respiratory infection	The proportion children under 5 who had a cough and difficulty breathing or a fever in the past 2 weeks, and for whom treatment was sought at a health facility or from a health provider	DHS: 1995, 2000–2001, 2006, 2011
Socioeconomic factors		
Household electricity	The proportion of households with functional electricity	Census: 1991, 2002
		DHS: 1995, 2000–2001, 2006, 2011
		MIS: 2009–2010
		AIS: 2011
		UNPS:2010–2011
Female headship of households	The proportion of households with a female head of household	Census: 1991, 2002
		DHS: 1995, 2000–2001, 2006, 2011
		MIS: 2009–2010
		AIS: 2011
		IHS: 1992–1993
		UNHS: 1999–2000, 2002–2003, 2005–2006, 2009–2010
		UNPS: 2009–2010, 2010–2011
		UNSDS: 2004, 2008
Household size	The mean number of members per household	Census: 1991, 2002
		DHS: 1995, 2000–2001, 2006, 2011
		MIS: 2009–2010
		AIS: 2011
		IHS: 1992–1993
		UNHS: 1999–2000, 2002–2003, 2005–2006, 2009–2010
		UNPS: 2009–2010, 2010–2011
		UNSDS: 2004, 2008
Educational attainment of women aged 15–44	The mean years of schooling for women aged 15–44	Census: 1991, 2002
		DHS: 1995, 2000–2001, 2006, 2011
		MIS: 2009–2010
		AIS: 2011
		IHS: 1992–1993
		UNHS: 1999–2000, 2002–2003, 2005–2006, 2009–2010
		UNPS: 2009–2010, 2010–2011
		UNSDS: 2004, 2008
Improved dwelling wall type	The proportion of households with dwelling walls constructed with an improved material	DHS: 1995, 2000–2001, 2006, 2011
		MIS: 2009–2010
		AIS: 2011
		UNHS: 1999–2000, 2002–2003, 2005–2006, 2009–2010
		UNPS: 2009–2010, 2010–2011
		UNSDS: 2004, 2008
Improved sanitation	The proportion of households with improved sanitation	DHS: 2006, 2011
		MIS: 2009–2010
		UNHS: 1999–2000, 2002–2003, 2005–2006, 2009–2010
		UNPS: 2009–2010, 2010–2011
		UNSDS: 2004, 2008
Improved water source	The proportion of households using an improved water source	DHS: 1995, 2000–2001, 2006, 2011
		MIS: 2009–2010
		AIS: 2011
		UNHS: 1999–2000, 2002–2003, 2005–2006, 2009–2010
		UNPS: 2009–2010, 2010–2011
		UNSDS: 2004, 2008

*DHS* Demographic and Health Survey, *MIS* Malaria Indicator Survey, *AIS* AIDS Indicator Survey, *Netmark* Netmark Survey, *IHS* Integrated Household Survey, *UNHS* Uganda National Household Survey, *UNPS* Uganda National Panel Survey, *UNSDS* Uganda National Service Delivery Survey, *Census* National Population Census

We included 20 MCH indicators that have previously been shown to be related to child survival [22] (Table 1): antenatal care (ANC, 1 and 4 visits); skilled birth attendance (SBA); immunization with the Bacillus Calmette-Guérin (BCG), measles, oral polio (three doses, OPV3), and pentavalent vaccines; exclusive breastfeeding (EBF); the receipt of at least two tetanus toxoid injections during pregnancy; reported use of oral rehydration salts for diarrhea; reported seeking of care at a health facility for children under 5 in response to suspected pneumonia; prevalence of childhood underweight and stunting; intermittent preventive therapy against malaria during pregnancy (IPTp, one and two doses); household ownership of ITNs; ITN use by children under 5; household receipt of IRS; the proportion of households that either owned at least one ITN or received IRS; the proportion of children under 5 who either slept under an ITN the night before the survey or lived in a household that received IRS; reported receipt of ACTs for children under 5 who had a fever in the last 2 weeks; and the proportion of ACT antimalarials prescribed for febrile children under 5. Additionally, we included seven socioeconomic covariates previously shown to be related to child health outcomes [23–26]: household size, female headship, electricity availability, improved wall type, improved sanitation, improved water source, and years of education for women aged 15–44. Due to data availability, we restricted our analysis to between 1990 and 2011.

We focus on the results For a subset of indicators in this paper, but trends for all indicators can be found in Additional file 1. We also produced national estimates by pooling microdata within and across surveys. All regional and national results can be downloaded from the Global Health Data Exchange (GHDx), a publicly available health data catalogue [27].

### Mapping

We report estimates for 10 sub-regions (Central 1, Central 2, Kampala, East Central, Eastern, North, Karamoja, West Nile, Western, and Southwest) delineated in the 2011 Demographic and Health Survey (DHS; Additional file 2) [28]. We sought to conduct our analyses at the district level, but experienced barriers to doing so appropriately. Uganda has frequently redistricted its administrative boundaries, creating new district boundaries and reformulating existing ones several times since 1990 (i.e. there were 44 districts in 1997, 79 in 2006, and 112 in 2010 [29, 30]). GPS coordinates were not available for every survey in this analysis, and since there was insufficient documentation of restricting activities, we could not consistently extract and reassign estimates to their corresponding district in 2010. As a result, we opted to use the 10 regions delineated in the 2011 DHS.

### Data processing

We calculated an annual series of under-5 mortality estimates for each region from surveys and censuses that included birth history modules (Table 1). We applied synthetic life table methods to pooled complete birth history data from sources where complete birth histories were collected (i.e. DHS) to generate direct estimates of under-5 mortality [15]. For sources where summary birth histories only were collected (i.e. AIDS Indicator Survey and both censuses), we applied the combined version of the maternal age cohort and maternal age period methods to generate indirect estimates of under-5 mortality [31].

We calculated regional intervention coverage estimates for each survey-year of available data. Aside from information extracted from Netmark survey reports (Table 1), all estimates were produced using microdata and accounting for each survey’s complex multistage design. For most child-level indicators, we included children aged between 0 and 59 months; however, we excluded children under 12 months of age for immunizations, except for BCG, due to their recommended dosing schedule [32]. For BCG immunization, however, we computed coverage estimates for two age groups – under 12 months and under 59 months – in order to both reflect timely BCG vaccination and overall protection among children under 5. We report on BCG immunization rates for the latter age group below, but additional information on BCG coverage estimation and corresponding results can be found in Additional files 1 and 3, respectively.

Most survey questions pertaining to ANC, SBA, and IPTp only covered the respondent’s most recent birth. As this restriction disproportionately excludes older children from high parity mothers, we restricted the age range for children aged 0–12 months to avoid bias. Additionally, the Uganda National Panel Survey (UNPS) only ascertained pentavalent and measles immunization status for children aged 0–24 months, and weight measurements were taken for children aged 6–59 months [33]. In order to maximize data inclusion while keeping age groups consistent across surveys, we used the UNPS age groups for all survey data extraction pertaining to these indicators. Immunization coverage estimates were obtained using vaccine cards whenever they were available; in cases where cards were not present at the time of survey, maternal recall was used to supplement immunization data.

### Estimation process for under-5 mortality

We adapted a previously described modeling and validation framework for modeling regional trends in under-5 mortality in Uganda [15]. Specifically, we applied the following model:

$$ logit\left(5q{0}_{i,t,s}\right)\left|{\theta}_{i,t,s}\right.,{\sigma}^2\sim Normal\left({\theta}_{i,t,s},{\sigma}^2\right) $$

$$ {\theta}_{i,t,s}={\beta}_0+{\beta}_1\cdot t+{\beta}_2\cdot {I}_{s\notin DHS}+{u}_i+{v}_i\cdot t+{w}_t+{\delta}_{i,t}+{\gamma}_{i,s} $$

where 5*q*0_*i*,*t*,*s*_ is under-5 mortality in region (*i*), year (*t*), as measured by source (*s*). The *β* terms are fixed effects; *β*_0_ and *β*_1_ are the global intercept and slope, respectively, and *β*_2_ is an adjustment coefficient included for non-DHS sources to account for an observed discrepancy between under-5 mortality estimates derived from DHS surveys and those derived from other surveys. All other terms are random effects. Specifically, *u*_*i*_ and *v*_*i*_ are a regional-level random intercept and slope, respectively, and are both assigned conditional autoregressive priors [34]; these terms allow for each region to deviate from the global level and linear trend in under-5 mortality. *w*_*t*_ is a year-level random intercept assigned a first-order random walk prior [35]; this term allows for non-linearity in the global time trend. Similarly, *δ*_*i*,*t*_ is a region-year level random intercept with the prior given by the interaction between a conditional autoregressive prior for spatial trends and a first order random walk prior for temporal trends [36]. This random effect allows for non-linearity in the region-specific time trends. Finally, *γ*_*i*,*s*_ is a source-year level random effect assigned an independent and identically distributed normal prior and is included to account for autocorrelation in estimates of under-5 mortality derived from the same source in the same region. Weakly informative normal priors were assigned to all fixed effects and weakly informative gamma priors were applied to the log precision of all random effects. To generate predictions from this model, we approximated the posterior distribution of *θ*_*i*,*t*_ by setting *I*_*s* ∉ *DHS*_ and *γ*_*i*,*s*_ to 0. The median, 2.5th, and 97.5th percentiles of this distribution were inverse-logit transformed to generate the point estimates and confidence intervals (CIs) for 5*q*0_*i*,*t*_ in each region and year.

### Estimation process for intervention coverage

We used a two-step modeling approach to generate regional trends from 1990 to 2011 for each indicator. In the first stage, we fit the following linear mixed-effects model with random intercepts and slopes for each region.

$$ g\left({y}_{it}\right)={\beta}_o+{\beta}_1{h}_{1t}+{\beta}_2{h}_{2t}+{\gamma}_{0i}+{\gamma}_{1i}{h}_{1t}+{\gamma}_{2i}{h}_{2t} $$

$$ g\left({y}_{it}\right)=\left\{\begin{array}{c}\hfill log it\left({y}_{it}\right),\; if\;{y}_{it}\in \left[0,1\right]\hfill \\ {}\hfill log\left({y}_{it}\right),\; if\;{y}_{it}\in \left[0,\infty \right)\;\hfill \end{array}\right. $$

Observations are indexed to region *i* and year *t*. For modeling coverage estimates, which are bounded between 0 and 1, logit transformation was applied. On the other hand, for variables such as years of maternal education log transformation was used. We used a one-knot natural cubic spline with two basis functions (*h*_*1*_ and *h*_*2*_) to act as a smoother. The random effects (*γ*_*0i*_, *γ*_*1i*_, and *γ*_*2i*_) allow the levels and trends to vary between regions.

In the second step, the predicted trend from this linear model acts as the mean prior for Gaussian process regression (GPR), which is implemented with a Matern covariance function [37, 38]. GPR is a nonparametric technique for interpolating non-linear trends that has been used extensively in the estimation of time series data [39–43]. Briefly, it takes into account the model variance as well as the relative sampling uncertainty of the observed data to estimate a posterior mean function. We generated trends with uncertainty for each indicator by drawing 1,000 times from the posterior distribution and back-transforming to the original scale. The point estimate was based on the median of the draws, and 95 % CIs were obtained by taking the 2.5th and 97.5th percentiles of the samples.

### Overall intervention coverage

We created two overall intervention coverage metrics to summarize regional intervention levels. First, we estimated an overall intervention coverage metric that included 11 indicators spanning the spectrum of interventions included in this analysis: the proportion of households with IRS, ITN ownership or both; IPTp2; self-reported receipt of ACTs after fever; EBF; BCG, measles, OPV3, and pentavalent immunization coverage; ANC4; SBA; and the proportion of children who were not underweight. When constructing overall coverage metrics, prior theory-based judgments may be incorporated to reflect the relative value of interventions [44]. For simplicity and ease of interpretation, we applied equal weights for each intervention included in the overall coverage metric, therefore defining overall intervention coverage as the average of the 11 interventions. We compared the overall intervention coverage trend to under-5 mortality and socioeconomic variables using Pearson correlation coefficients.

### Software

All analyses were conducted using Stata 13.1 (StataCorp, College Station, TX) and R version 3.0.1 (R Foundation for Statistical Computing, Vienna, Austria). The model used for mortality estimation was fit using the INLA package [45].

### Ethical approval

Permission to implement this research project was obtained from the Ministry of Health of Uganda. Ethical approval for this study was obtained from the institutional review board of the University of Washington, Uganda National Council of Science and Technology, and School of Medicine Research Ethics Committee. The study was conducted in compliance with national regulatory and ethics guidelines.

## Results

### Under-5 mortality

**Fig. 1:**
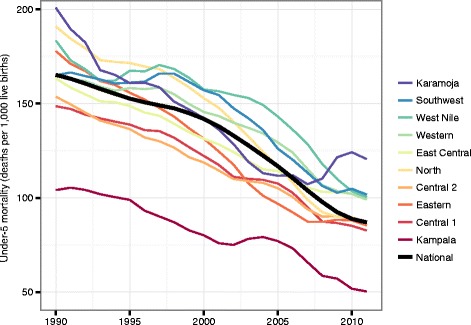
Regional trends in under-5 mortality, 1990–2011

We found large declines in under-5 mortality for all regions since 1990 (Fig. 1). The national rate of decline has quickened since 2000, with a 39 % decrease between 2000 and 2011 compared to a 14 % decrease from 1990 to 2000. However, subnational estimates revealed considerable regional variation. Kampala experienced consistently lower mortality than all other regions between 1990 and 2011, although the gap between Kampala and the rest of the country declined. In 1990, Karamoja (201 deaths per 1,000 live births; 95 % CI, 169–236), North (191 deaths per 1,000 live births; 95 % CI, 164–221), and West Nile (184 deaths per 1,000 live births; 95 % CI, 154–217) had the highest mortality. These regions saw steep declines between 1990 and 2011, especially in the North region (a 54 % decrease). Despite large decreases in under-5 mortality from 1990 to 2007, Karamoja showed an increase in rates between 2007 (107 deaths per 1,000 live births; 95 % CI, 88–130) and 2010 (124 deaths per 1,000 live births; 95 % CI, 100–153), although the uncertainty intervals were wide.

### Childhood underweight

**Fig. 2:**
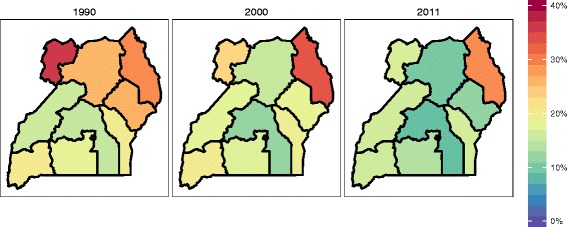
Prevalence of underweight among children under 5, 1990–2011

The prevalence of childhood underweight declined in Uganda, falling from 20 % (95 % CI, 16–26 %) in 1990 to 14 % (95 % CI, 12–15 %) in 2011 (Fig. 2). Since 1990, a number of regions recorded even faster declines in childhood underweight, dropping 14 percentage points in the Eastern region, 16 percentage points in North, and 19 percentage points in West Nile. By contrast, levels of childhood underweight largely stagnated in Karamoja and Western regions since 1990. Further, Karamoja’s rates of childhood underweight, 29 % (95 % CI, 24–34 %) in 2011, remained persistently higher than in the rest of Uganda.

### Malaria interventions

**Fig. 3:**
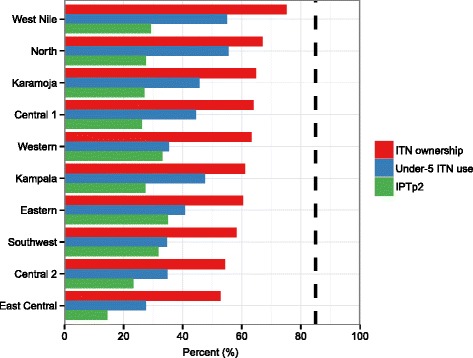
Regional malaria intervention coverage, 2011. Note: Dotted line indicates National Malaria Control Programme coverage target of 85 % for 2010

Uganda has rapidly scaled up coverage of several malaria interventions. Prior to 2004, all regions recorded coverage levels below 10 % for ITNs and ACTs. By 2011, national rates of ITN ownership reached 59 % (95 % CI, 36–79 %) and self-reported receipt of ACTs for febrile children under 5 increased to 49 % (95 % CI, 34–65 %). Despite these gains, no region met the National Malaria Control Programme (NMCP) target of 85 % by 2010 for ITN ownership, under-5 ITN use, or IPTp2 (Fig. 3) [46]. In addition, IPTp2 remained quite low across regions (29 % in 2011), a striking finding since IPTp2, which is prescribed at ANC visits, consistently lagged behind levels of ANC4. Nationally, IPTp2 coverage was 18 percentage points lower than ANC4, and only one region (Eastern) had IPTp2 levels (35 %; 95 % CI, 21–54 %) that were comparable to ANC4 (35 %; 95 % CI, 24–47 %) in 2011.

### Childhood immunizations

BCG and measles vaccine coverage increased in all regions from 1990 to 2011. Nationally, BCG immunization coverage rose from 78 % (95 % CI, 62–89 %) in 1990 to 94 % (95 % CI, 89–97 %) in 2011, and measles vaccination climbed from 63 % (95 % CI, 39–82 %) to 85 % (95 % CI, 75–91 %) during this time. In contrast, OPV3 coverage remained relatively unchanged across regions, increasing slightly from 1990 to 2000 and then somewhat declining since 2000. Pentavalent immunization coverage (77 %; 95 % CI, 51–92 %) reached comparable levels to OPV3 coverage in 2011 (76 %; 95 % CI, 52–90 %) demonstrating a rapid scale-up since its introduction in 2002. At the regional level, immunization coverage was more varied. In 2011, OPV3 coverage ranged from 69 % (95 % CI, 44–87 %) in Central 1 to 83 % (95 % CI, 63–93 %) in Southwest, and pentavalent vaccination coverage spanned from 67 % (95 % CI, 42–86 %) in East Central to 86 % (95 % CI, 69–95 %) in Southwest. In general, Central 1 had lower levels of immunization coverage than the other regions, whereas Southwest had higher vaccination rates.

### Other key MCH indicators

**Fig. 4:**
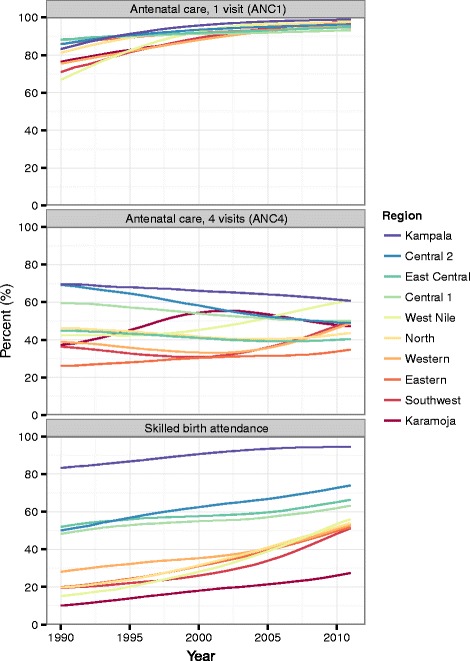
Regional trends in antenatal care (1 and 4 visits) and skilled birth attendance, 1990–2011

Between 1990 and 2011, ANC1 coverage increased across regions, narrowing the gap between the regions with the highest and lowest levels of ANC1 over time (Fig. 4). In 1990, ANC1 coverage ranged from 67 % (95 % CI, 40–87 %) in West Nile to 88 % (95 % CI, 70–96 %) in East Central, a difference of 21 percentage points; more than two decades later, this coverage gap shrunk to six percentage points, with the ANC1 coverage range spanning 93 % (95 % CI, 85–97 %) in Central 1 to 99 % (95 % CI, 98–100 %) in Kampala. Similar gains were not observed for ANC4 coverage, with the national average remaining relatively unchanged (49 %; 95 % CI, 30–65 % in 1990 and 47 %; 95 % CI, 41–54 % in 2011). Regionally, ANC4 trends diverged, with several regions experiencing decreasing ANC4 coverage at the same time as others showed gradual gains in coverage. By contrast, SBA coverage increased substantially for a number of regions since 1990, particularly in North (a 34 percentage point increase), West Nile (a 41 percentage point increase), Eastern (a 32 percentage point increase), and Southwest (a 32 percentage point increase). SBA coverage still remained quite varied across regions, with Kampala and nearby regions experiencing much higher rates (SBA coverage was 95 %; 95 % CI, 90–97 % in Kampala) than Karamoja (27 %; 95 % CI, 16–43 %). Nationwide, EBF moderately increased, rising from 54 % (95 % CI, 36–71 %) in 1990 to 61 % (95 % CI, 54–67 %) in 2011. By 2011, Karamoja had the highest rates of EBF, at 73 % (95 % CI, 58–84 %), whereas Kampala recorded the lowest levels (46 %; 95 % CI, 31–62 %).

### Overall intervention coverage

**Fig. 5:**
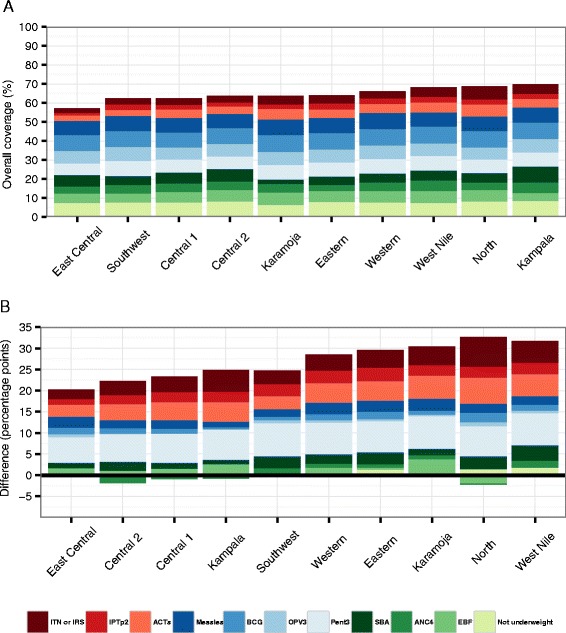
Overall intervention coverage in 2011 (**a**) and change in overall intervention coverage from 1990 to 2011 (**b**)

Uganda experienced a large increase in overall intervention coverage since 2000, which was largely driven by gains in coverage of malaria vector control (ITNs or IRS) and pentavalent immunization (Fig. 5). In 2011, Kampala recorded the highest level of overall intervention coverage at 70 % whereas East Central had the lowest at 57 %. West Nile and North recorded the largest gains in overall intervention coverage since 1990, at 31 and 30 percentage points, respectively. Overall coverage was strongly correlated with under-5 mortality (ρ = −0.85), but its relationships with measures of education (ρ = 0.57) and other socioeconomic indicators (with ρ ranging from 0.42 to 0.50) were more moderate. The relationship between under-5 mortality and overall intervention coverage was stronger than the correlation between under-5 mortality and education (ρ = −0.77), as well as under-5 mortality and other socioeconomic indicators (with ρ ranging from −0.50 to −0.75).

## Discussion

This study represents the first comprehensive regional assessment of levels and trends of MCH indicators and socioeconomic factors in Uganda. All regions have achieved substantial progress in reducing under-5 mortality as well as scaling up coverage of malaria control interventions and pentavalent vaccination. At the same time, coverage of other key MCH interventions, such as OPV3 and ANC4, have generally flattened or declined over time. Analyzing regional trends revealed marked heterogeneity and geographic disparities in both coverage levels and outcomes, emphasizing the importance of routinely monitoring local health needs in a timely manner. Subnational benchmarking of health intervention coverage is a crucial step in identifying areas to which resources should be directed in Uganda and sub-Saharan Africa.

Although under-5 mortality decreased throughout Uganda, geographic inequalities persisted, with Kampala consistently recording much lower rates of under-5 deaths than other regions from 1990 to 2011. Further, progress in reducing child mortality in Karamoja stalled after 2007, and its prevalence of childhood underweight was the highest in Uganda for 2011, more than 10 percentage points higher than the next-highest region. Although Karamoja achieved high levels of coverage for some interventions, particularly EBF and pentavalent vaccination, its health outcomes lagged behind other regions. Food insecurity due to climactic shocks, disease, and conflict is well-documented in this region [47–53], which is largely populated by pastoralist groups. Heavy flooding in 2007 reportedly devastated crops and isolated many parts of the region from food supplies [54], potentially contributing to the region’s high levels of child mortality. More recent efforts to improve Karamoja’s challenges with nutrition, such as food distribution initiatives led by organizations like the World Food Programme [55], may have resulted in improved child health outcomes. However, focused tracking of subnational trends and such targeted interventions will be necessary to optimally understand the impact of these efforts. Health policies and programs well-suited for some communities do not necessarily translate into the same health benefits in others; for instance, researchers found western areas of Uganda experienced high rates of malnutrition, in spite having adequate food production [56], and thus recommended programs focused on community nutrition and health education beyond food distribution. Further investigation into these strategies, ideally led by a combination of researchers and program leaders in Uganda (e.g. the 2013 Nutrition Surveillance Analysis for Karamoja [57]), could shed light on which types of programs work to address the country’s local malnutrition burdens.

Uganda’s trends along the maternal health continuum of care unveiled a number of notable findings. While all regions reached high levels of ANC1 coverage by 2011, trends for ANC4 coverage, which is recommended by Uganda’s national guidelines, were much more variable across regions [58]. This coverage gap aligns with reports of women starting ANC later points during their pregnancies [59, 60], making it challenging to complete four ANC visits before delivery. While levels of SBA increased across all regions, in 2011 there remained a wide gap between the highest performer (Kampala, at 95 %) and the lowest performer (Karamoja, at 27 %). Differences in SBA might be explained by educational attainment, health facility proximity, workforce shortages, or cultural preferences to deliver at home or with traditional birth attendants [61–63]. These disparities along a continuum of care for maternal health services are not unique to Uganda [17, 64] and point to two related MCH service delivery challenges facing many countries in sub-Saharan Africa: (1) increasing women’s use of ANC prior to giving birth; and (2) promoting heightened demand for delivery by skilled attendants and/or a stronger capacity for delivering in health facilities. Past work has shown that a number of factors hinder the provision of ANC and SBA, including distance to care [65], cost [61, 66], cultural practices [67], insufficient knowledge about the importance of ANC [67], and inadequate availability of medical supplies and equipment at facilities [68, 69]. Local health offices may consider introducing or scaling up current programs which include a combination of outreach activities led by community health workers [65], addressing gaps in facility-based resources to provide ANC and support routine deliveries, and incentive schemes, such as vouchers or conditional cash transfers, that address financial barriers and/or link ANC services to post-natal care [66, 70–72].

Uganda has substantially increased coverage of malaria control interventions since 2000, reflecting investments in ITN distribution, subsidizing costs of ITNs and ACTs, and expanding IRS activities [8, 73, 74]. While no region achieved the 2010 NMCP target of reaching 85 % coverage for ITN ownership, ITN use, and IPTp2, it is likely that a 2013 mass distribution campaign has bolstered ITN ownership and use in more recent years [75]. Routine monitoring of malaria control implementation at local levels, as well as the timely investigation into stagnating intervention coverage, are critical to maximizing malaria control impact in Uganda, a country that still experiences some of the highest rates of transmission in the world [76].

Most regions had lower levels of IPTp2 coverage than ANC4, which suggests there is a substantial constraint to receiving IPTp at ANC visits as recommended by Uganda’s national guidelines [46]. A nationally-representative survey of health facilities found that over 90 % of facilities in Uganda had sulfadoxine/pyrimethamine in stock in 2012 [67], indicating that stock-outs were an unlikely root cause of low IPTp2. Previous work suggests that prescription practices by health providers, including the misuse of sulfadoxine/pyrimethamine to treat clinical malaria, may be the main driver of low rates of IPTp [77, 78]. Further examination is needed to identify and address the factors leading to low IPTp2 coverage throughout Uganda.

Immunization progress has been variable in Uganda. Coverage of BCG and measles vaccination has increased markedly since 1990, while trends in polio and pentavalent coverage were more variable. The Reaching Every District approach promoted by the World Health Organization (WHO) sets a coverage target of 80 % for all districts in low- and middle-income countries in order to help rectify geographic inequities [79]. While this analysis was conducted at the regional level, the Reaching Every District target still provides a useful benchmark. Immunization coverage in 2011 exceeded 80 % for BCG and measles in all regions; however, only five and two regions achieved coverage over 80 % for OPV3 and pentavalent, respectively. Previous studies have attributed the rise in measles coverage, which has occurred in all regions, to expanded control efforts from 1999 to 2006 [80–82]. The gains in coverage could explain the nearly four-fold drop in under-5 measles deaths from 1990 to 2013 [40]. At the same time, OPV3 coverage has declined in all regions since 2000. While Uganda was certified as polio-free in 2006, wild poliovirus cases were confirmed in Amuru District (North) in 2009 and Bugiri District (East Central) in 2010 [83]. Notably, the North region had the third-lowest OPV3 coverage in 2009 (73 %) and the East Central region had the second-lowest coverage in 2010 (72 %). Subsequently, large-scale supplementary polio immunization campaigns were conducted in eastern and northern Uganda. In addition, a nationwide polio immunization campaign started in January 2015 [84]. While we were not able to estimate coverage trends beyond 2011, future benchmarking exercises using more recent data may help evaluate the results of these campaigns.

The quick rise of pentavalent vaccination coverage to similar levels as OPV3 in all regions demonstrates the successful implementation of the 2002 Gavi Vaccine Introduction Grant [85]. This scale-up is promising for more recent efforts in Uganda to introduce the pneumococcal conjugate vaccine, rotavirus vaccine, human papillomavirus vaccine, and inactivated polio vaccine. However, pentavalent coverage stagnated or declined since 2008 in most regions. Gavi ISS was suspended from 2006 to 2012, which may be related to the drops in coverage. Further research is needed to determine how to further improve routine immunization services in Uganda.

This study revealed substantial heterogeneity in health indicators and outcomes across Uganda’s regions. The decentralization of Uganda’s health system has emphasized management by local governance [86], which requires subnational benchmarking to optimally evaluate performance of health service delivery. While this work relied on household surveys and censuses, further efforts to improve the accuracy and timeliness of HMIS reporting in Uganda will greatly improve local evaluation efforts and guide resource allocation decisions [87]. Other subnational benchmarking studies, such as those conducted in Mexico [44, 88], have strengthened government accountability and supported the use of locally-relevant evidence to inform decision-making. The systematic tracking of local MCH outcomes and interventions is increasingly occurring in sub-Saharan Africa, as demonstrated by recent work in Zambia and Nigeria [17, 61], and investments by development partners like Gavi to support subnational assessments of immunization coverage [89]. In tandem, research producers and evidence users are working together to make these local data both more accessible and actionable, creating public data catalogues such as the GHDx [27] and WHO’s Global Health Observatory [90] and developing interactive, locally-focused data visualization tools, such as the Nigeria Health Map [91]. It is through these collaborative efforts, from the generation of subnational data to its dissemination, that local health evidence can be more effectively used by policymakers, program leaders, and funding organizations to guide their decision-making needs.

### Limitations

Our findings need to be interpreted within the context of some study limitations. First, a number of relevant interventions could not be analyzed due to limited data availability. For instance, we were unable to obtain reliable data for HIV interventions such as prevention of mother-to-child transmission of HIV or pediatric HIV treatment. Second, several MCH indicators were limited by question comparability across surveys. Notably, questions concerning EBF only considered feeding in the day prior to the survey instead of in the first six months of life [92], and ACT use and healthcare-seeking at health facilities were based on self-reported symptoms of fever and cough, respectively, rather than confirmed malaria or pneumonia diagnoses. Third, some of the data sources from the early 1990s, including the 1991 census and the 1995 and 2000–2001 DHS surveys, excluded certain districts in the North region from the sampling frame due to insecurity. It is possible that the point estimates from these surveys are biased; however, previous analysis using the 2006 DHS found that excluding the districts not sampled in the earlier surveys had little effect on coverage estimates [93]. Finally, most estimates from household surveys were derived from respondent recall, which can be imprecise and subject to bias. Nevertheless, we believe that by combining all available data sources, these findings constitute the best available estimates of levels and trends of key MCH interventions in Uganda.

## Conclusions

Uganda has experienced substantial declines in under-5 mortality and gains in ITN ownership and use, the receipt of ACTs, measles vaccination, ANC1, and SBA. However, progress in coverage of other indicators such as ANC4, IPTp2, and OPV3 has stalled. National level trends masked marked subnational heterogeneity, and further investigation is necessary to understand the drivers of regional variation. Enhancing the scope and reliability of health information systems would further enable the regular monitoring of levels and trends. Additional subnational benchmarking analyses, ideally at the administrative level where health services are delivered, should be conducted routinely in order to systematically guide resource allocation and policy decisions in Uganda and other countries in sub-Saharan Africa.

## Additional files

Additional file 1:
**Maps of indicator trends by region.** (DOCX 10443 kb) Additional file 2:
**Demographic and Health Survey (DHS) 2011 region boundaries.** (DOCX 57 kb) Additional file 3:
**Comparing BCG immunization coverage across age groups by region.** (DOCX 354 kb)

## Abbreviations

ACT: Artemisinin-based combination therapy
ANC: Antenatal care
BCG: Bacillus Calmette-Guérin
CIs: Confidence intervals
DHS: Demographic and Health Survey
EBF: Exclusive breastfeeding
GHDx: Global Health Data Exchange
GPR: Gaussian process regression
HMIS: Health Management Information System
IPTp: Intermittent preventive therapy during pregnancy
IRS: Indoor residual spraying
ISS: Immunization services support
ITN: Insecticide-treated net
MCH: Maternal and child health
NMCP: National Malaria Control Programme
OPV: Oral polio vaccine
SBA: Skilled birth attendance
UNPS: Uganda National Panel Survey
WHO: World Health Organization
