# Supplementary material for: Benchmarking health system performance across regions in Uganda: a systematic analysis of levels and trends in key maternal and child health interventions, 1990–2011
Source: BMC Med. 2015 Dec 3;13:285. doi: 10.1186/s12916-015-0518-x (PMC4668680; doi:10.1186/s12916-015-0518-x)
Supplement: Additional file 1: — Maps of indicator trends by region. (DOCX 10443 kb) [file 12916_2015_518_MOESM1_ESM.docx]

**Additional file 1**

**Figure S2: Trends for indicators, 1990, 2000, and 2011**

**Under-5 mortality (deaths per 1,000 live births)**

**
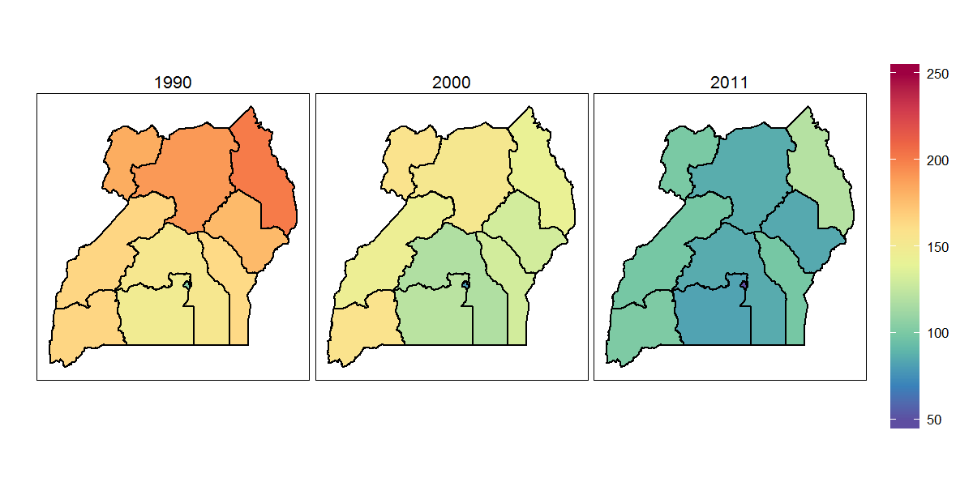
Antenatal care (1 visit)**

**
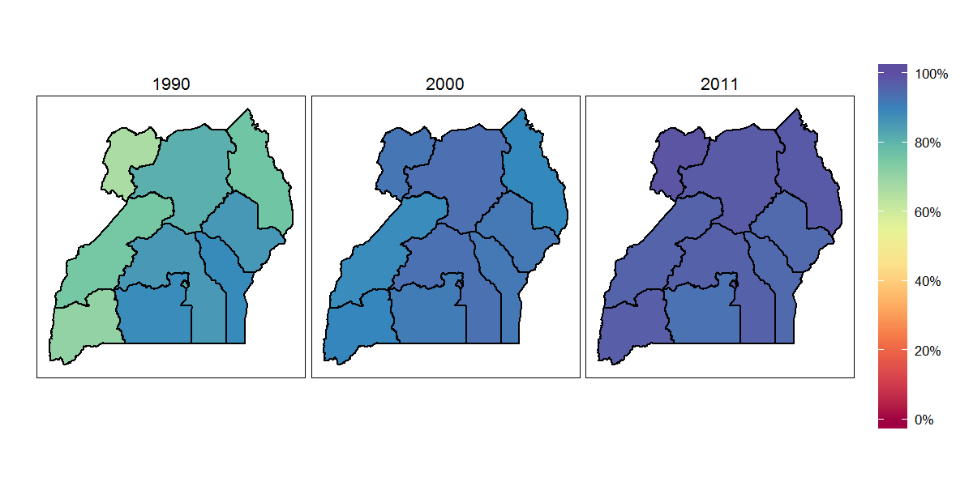
**

**Antenatal care (4 visits)**

**
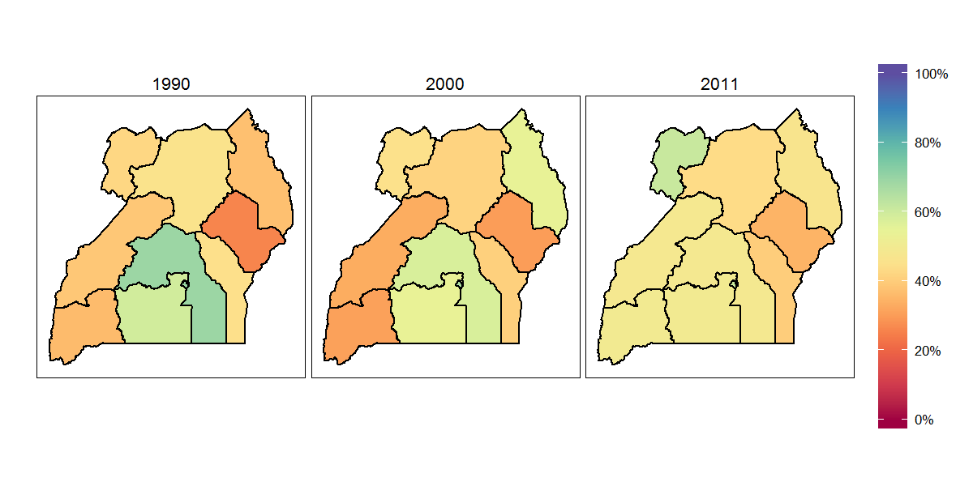
**

**Skilled birth attendance**

**
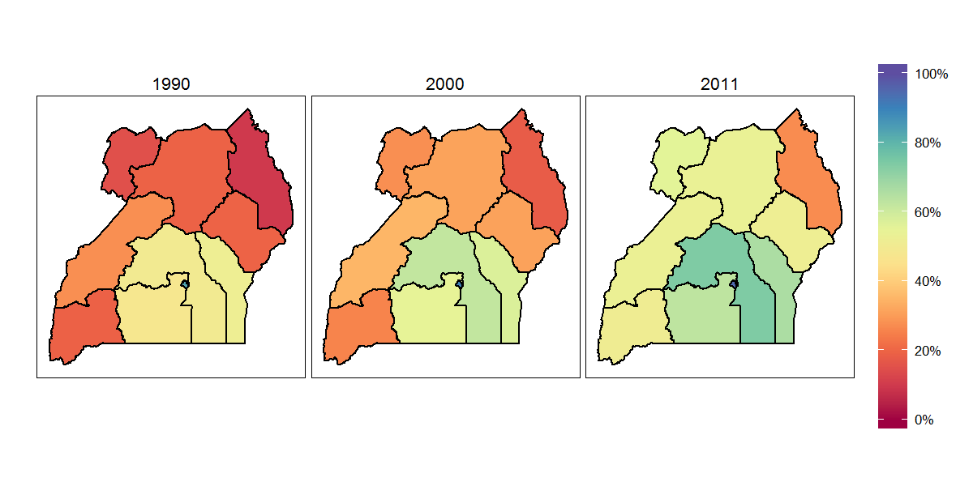
**

**BCG immunization (1 dose), children aged 0 to 12 months**

**
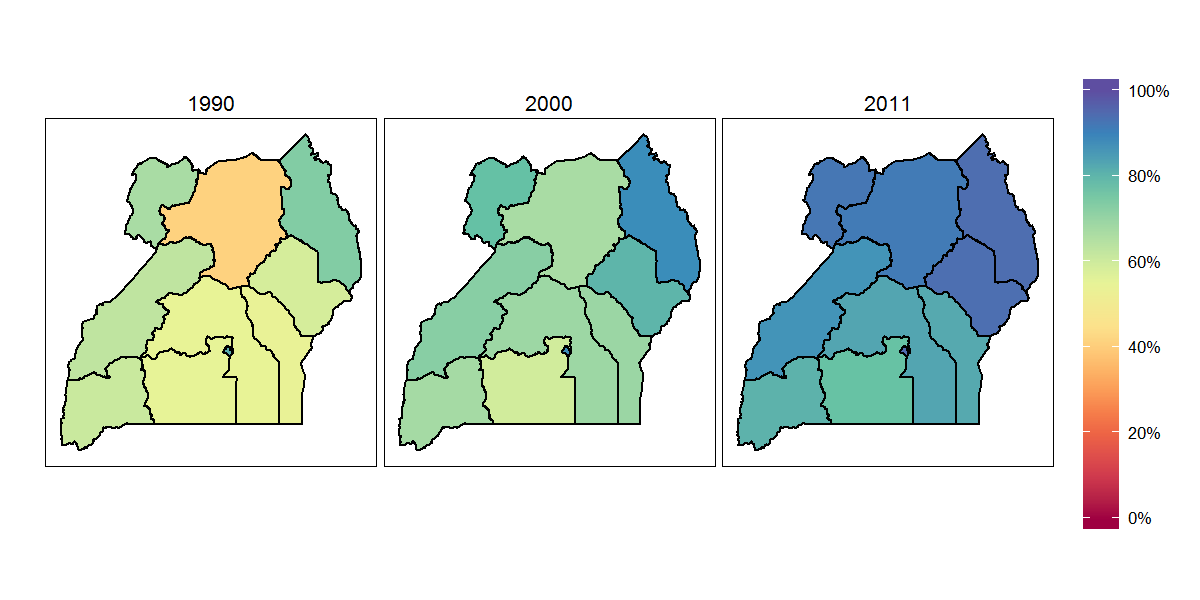
**

**BCG immunization (1 dose), children aged 0 to 59 months**

**
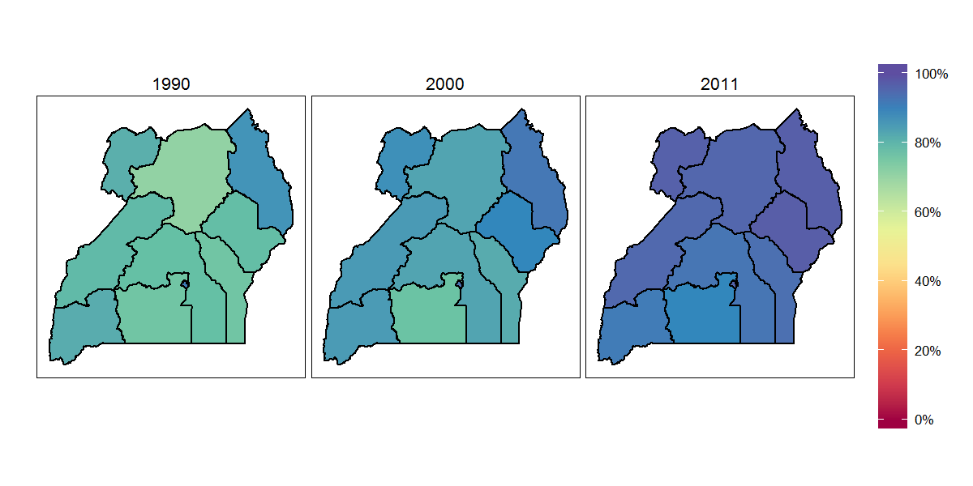
**

**Measles immunization (1 dose)**

**
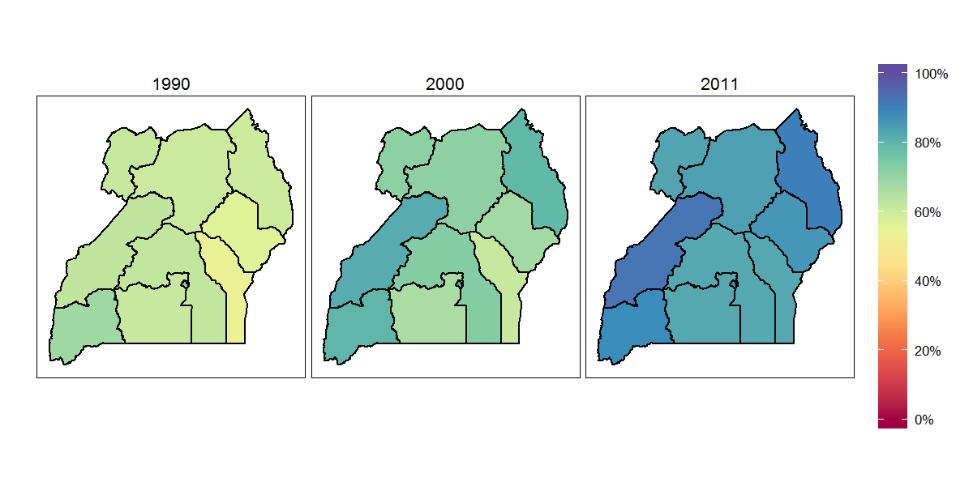
**

**Polio immunization (3 doses)**

**
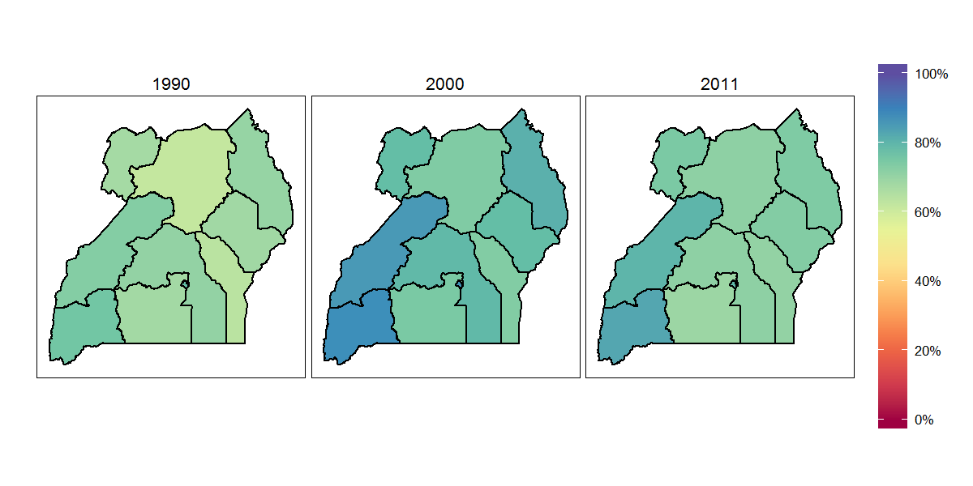
**

**Pentavalent immunization (3 doses)**

**
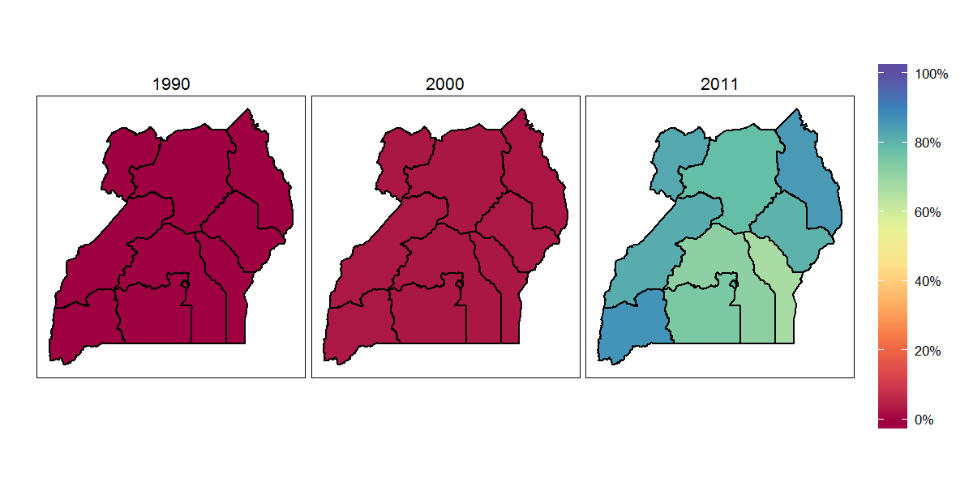
**

**Exclusive breastfeeding**

**
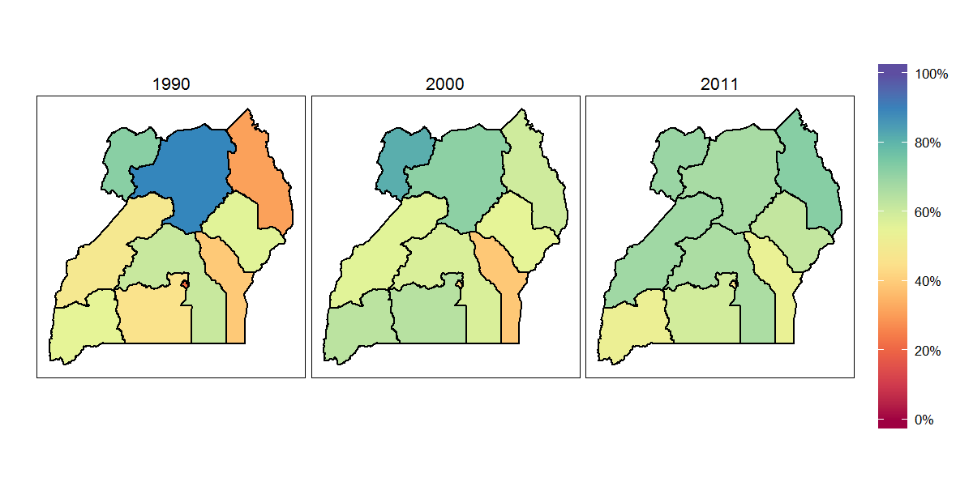
**

**Maternal immunization against tetanus (2 doses)**

**
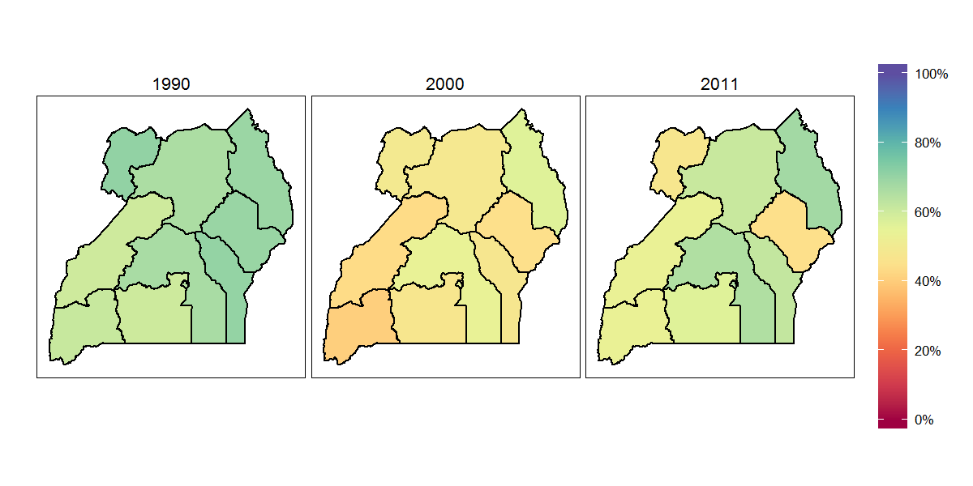
**

**Oral rehydration salts after diarrhea**

**
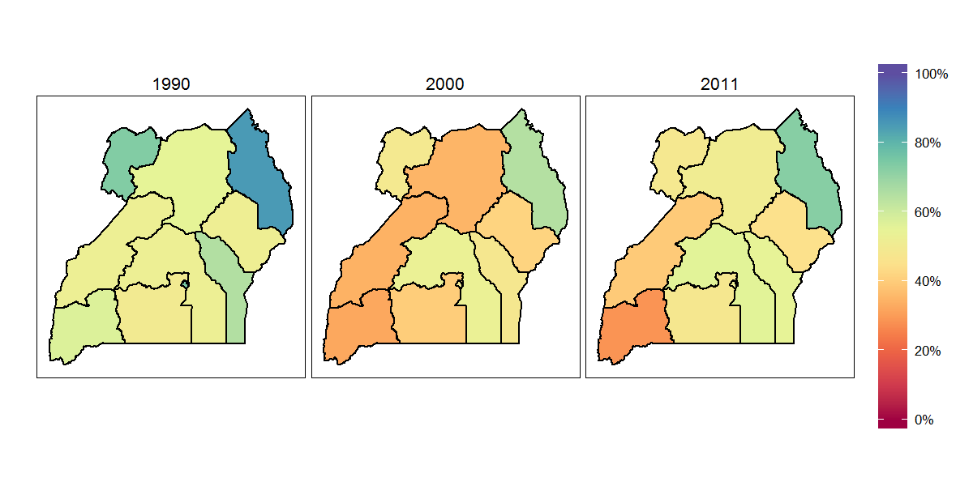
**

**Care at health facility after suspected acute respiratory infection**

**
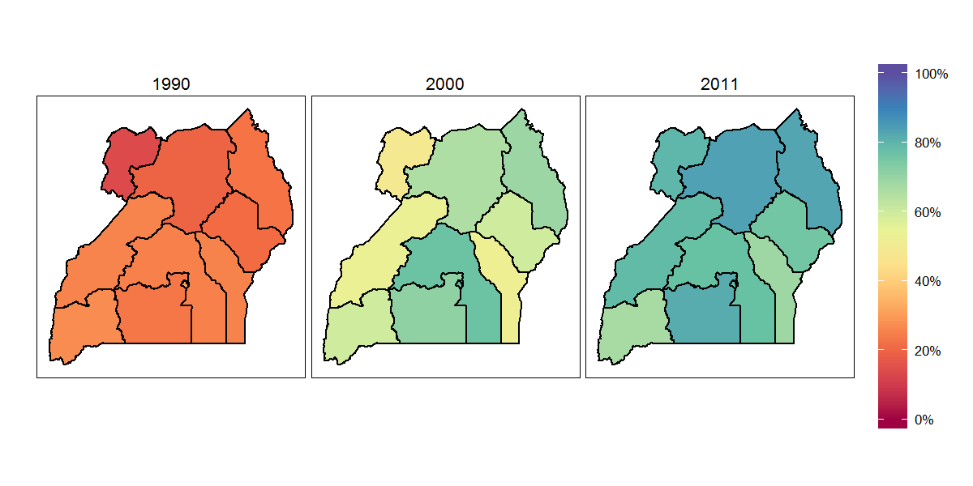
**

**Percentage of children underweight**

**
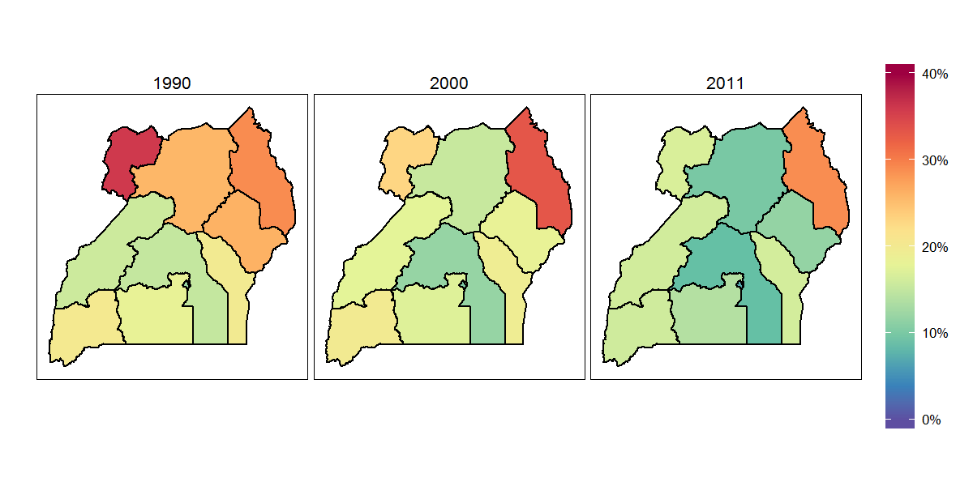
**

**Percentage of children stunted**

**
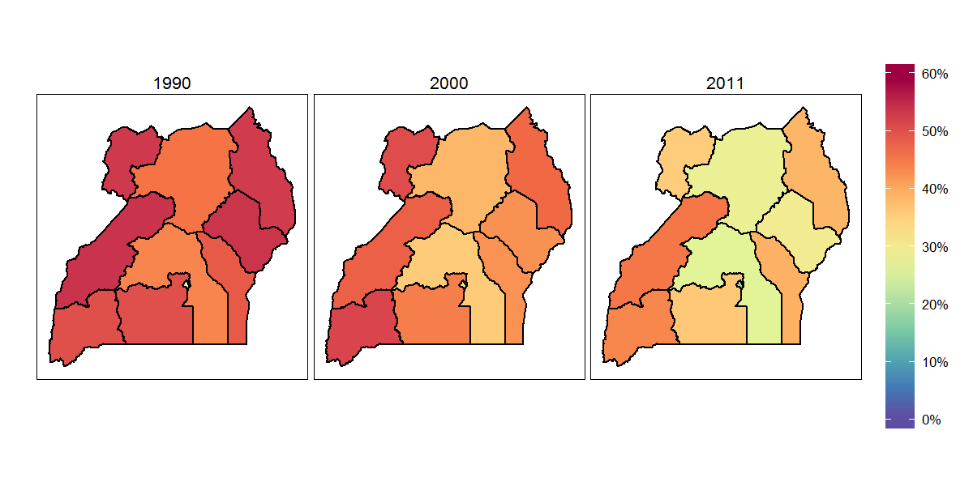
**

**Intermittent preventive therapy for malaria during pregnancy (1 dose)**

**
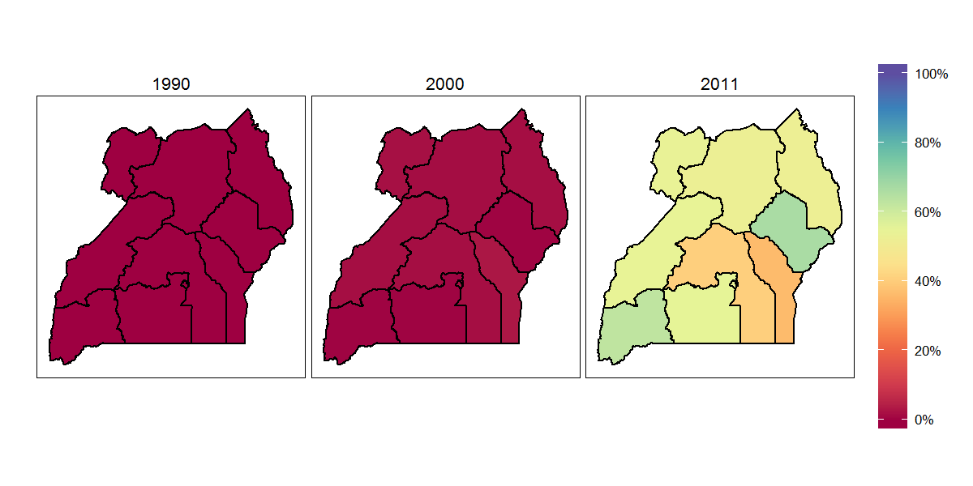
**

**Intermittent preventive therapy for malaria during pregnancy (2 doses)**

**
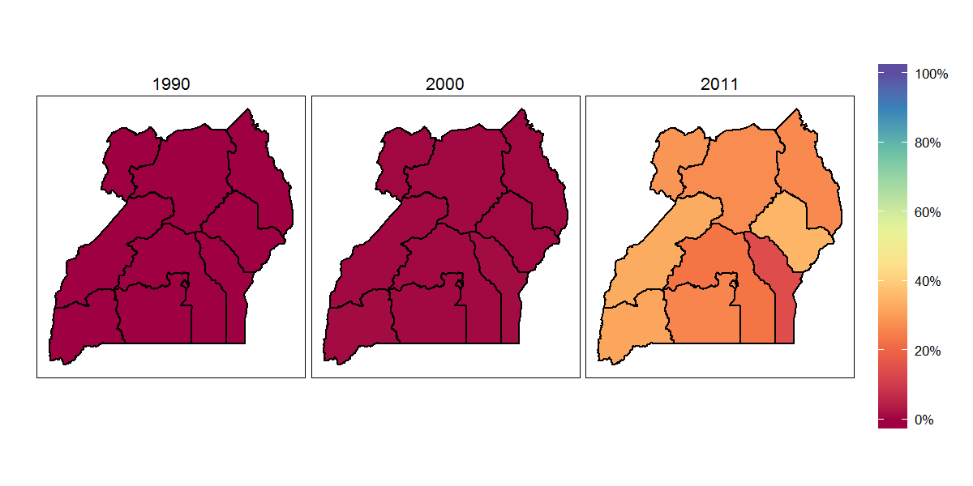
**

**Insecticide-treated net ownership**

**
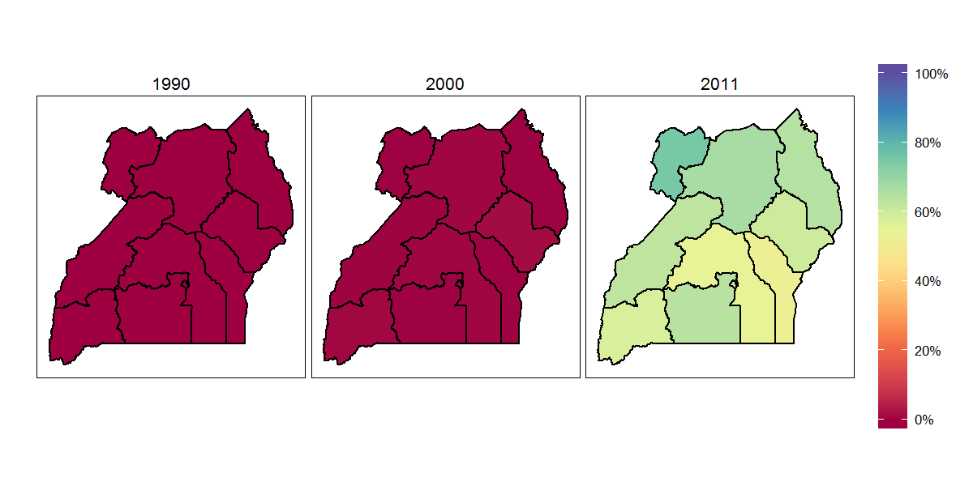
**

**Insecticide-treated net use by children under 5**

**
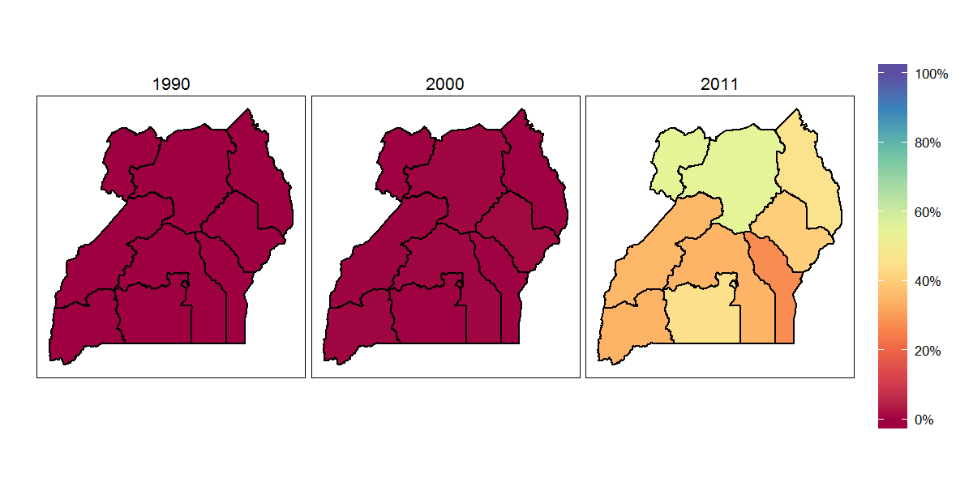
**

**Indoor residual spraying**

**
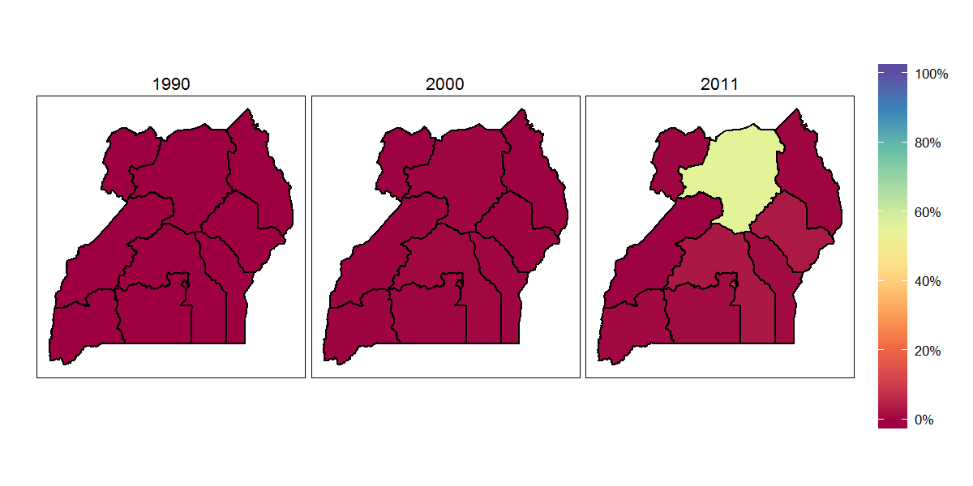
**

**Insecticide-treated net ownership or indoor residual spraying**

**
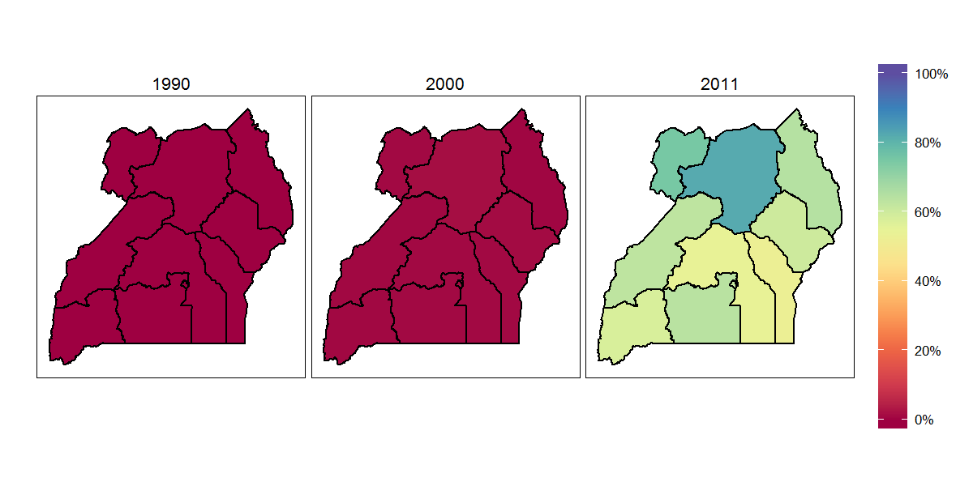
**

**Insecticide-treated net use by children under 5 or indoor residual spraying**

**
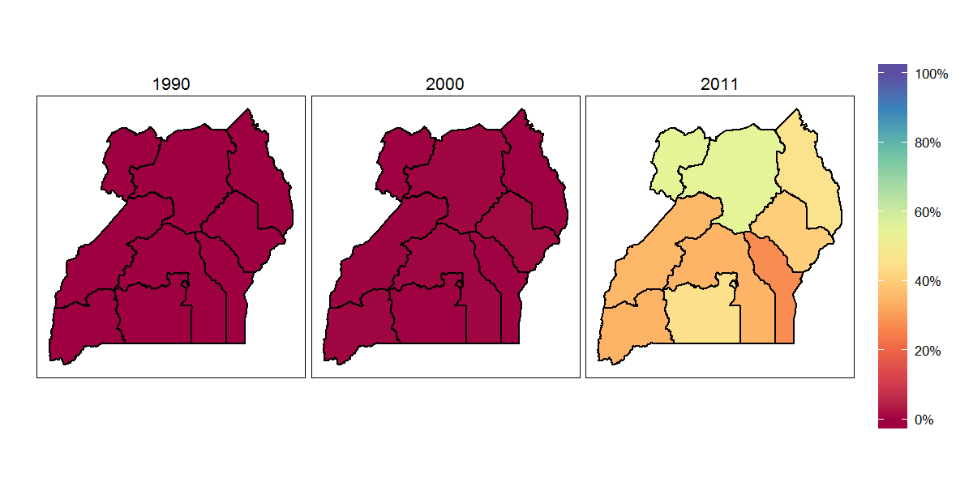
**

**Receipt of ACTs for febrile children under 5**

**
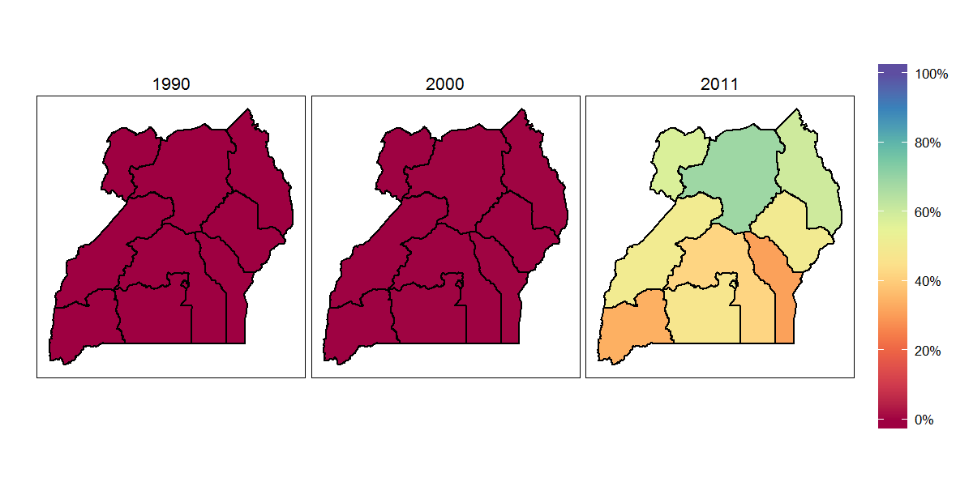
**

**Percent of antimalarials given to febrile children under 5 that were ACTs**

**
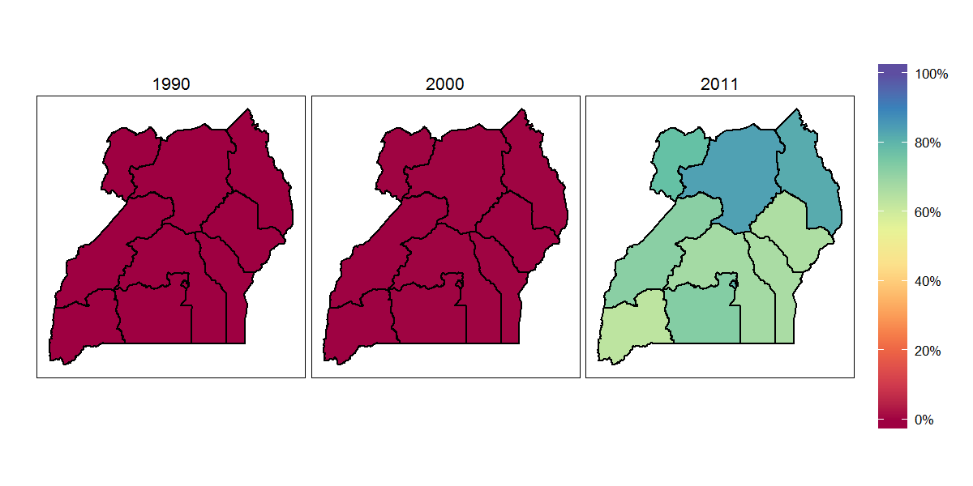
**

**Percentage of households with electricity**

**
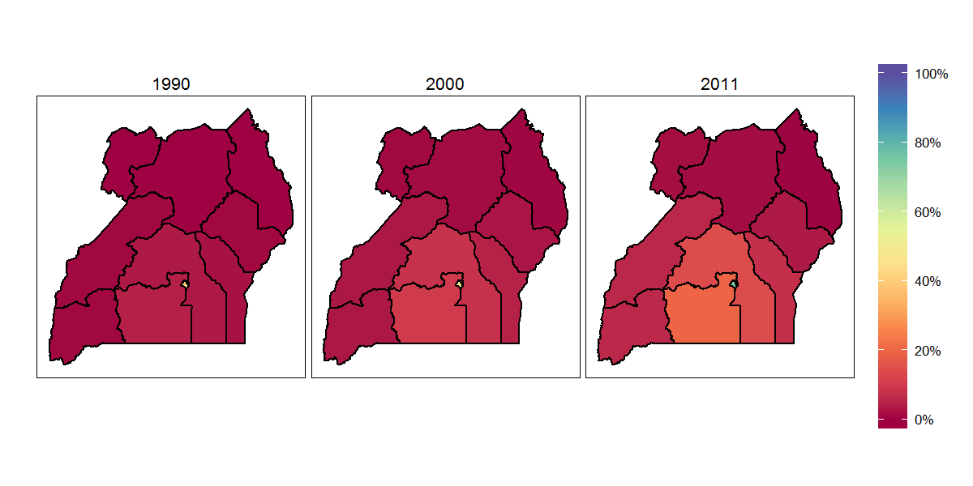
**

**Female headship of households**

**
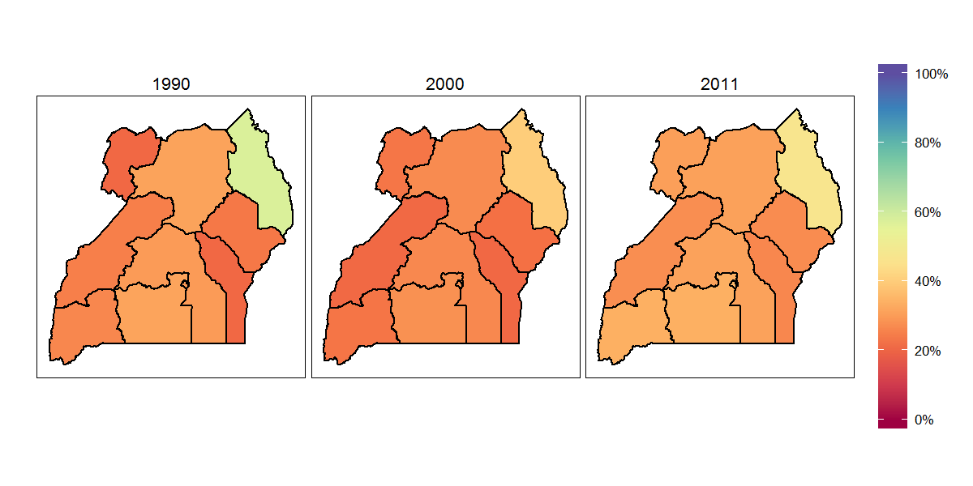
**

**Household size**

**
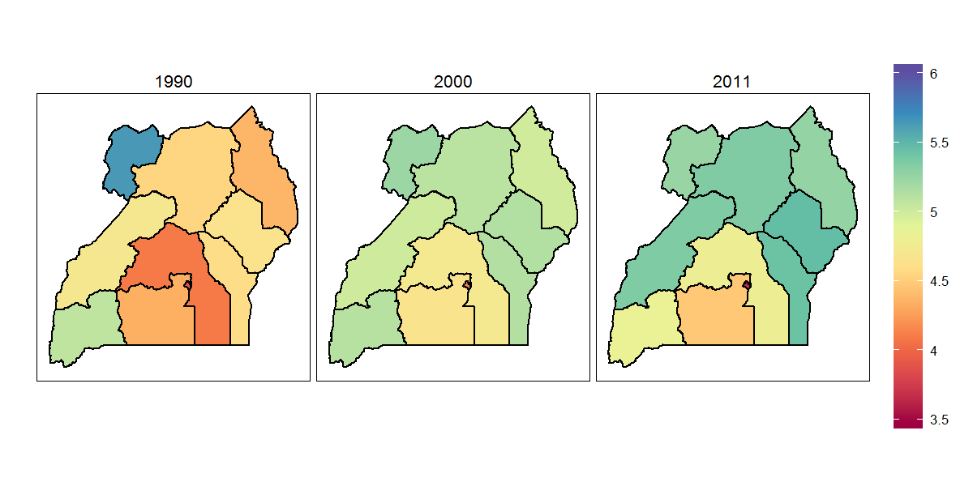
**

**Years of education of women 15 to 44 years old**

**
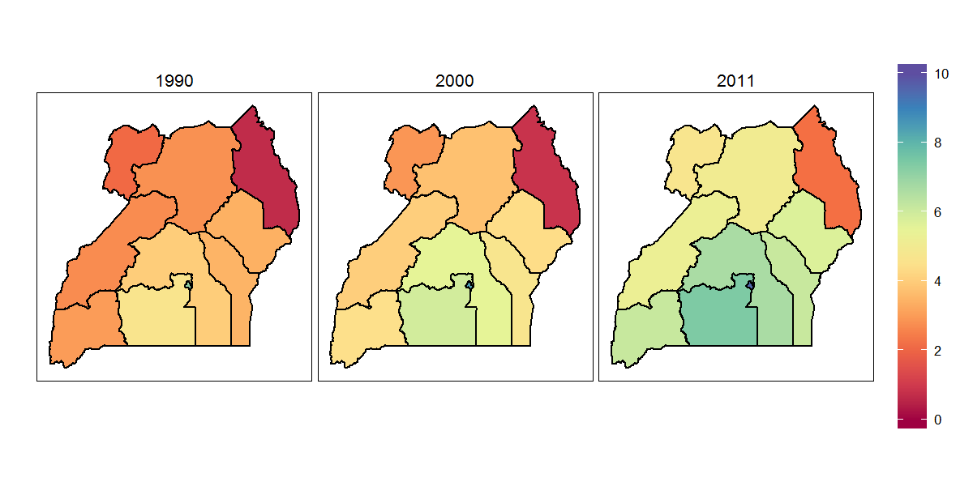
**

**Percentage of households with improved dwelling wall type**

**
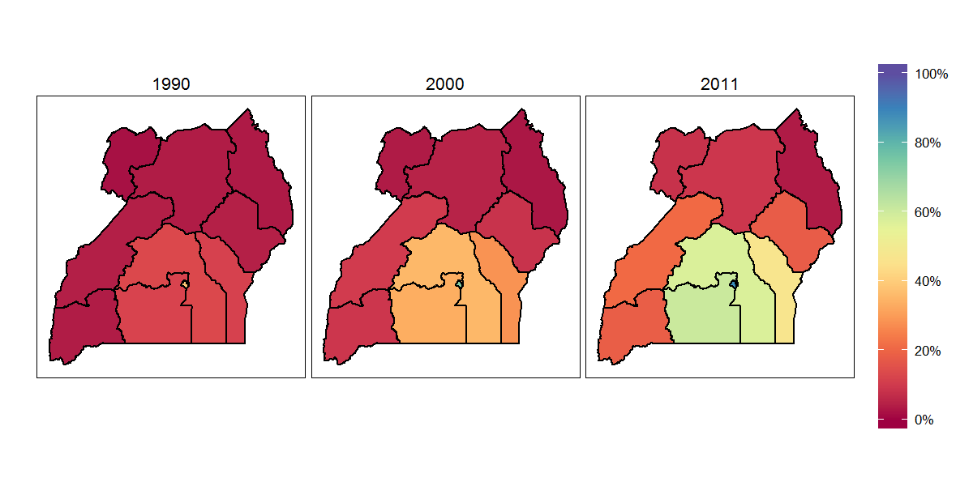
**

**Percentage of households with improved sanitation**

**
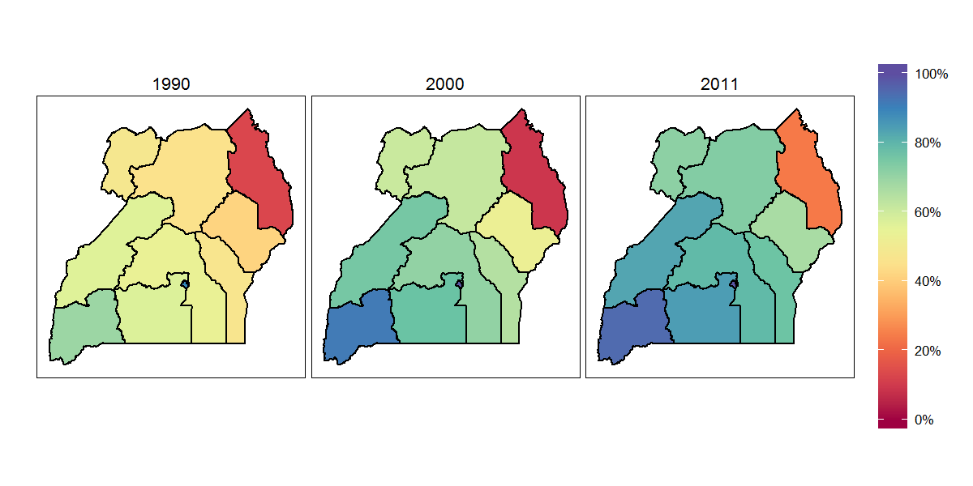
**

**Percentage of households with an improved water source**

**
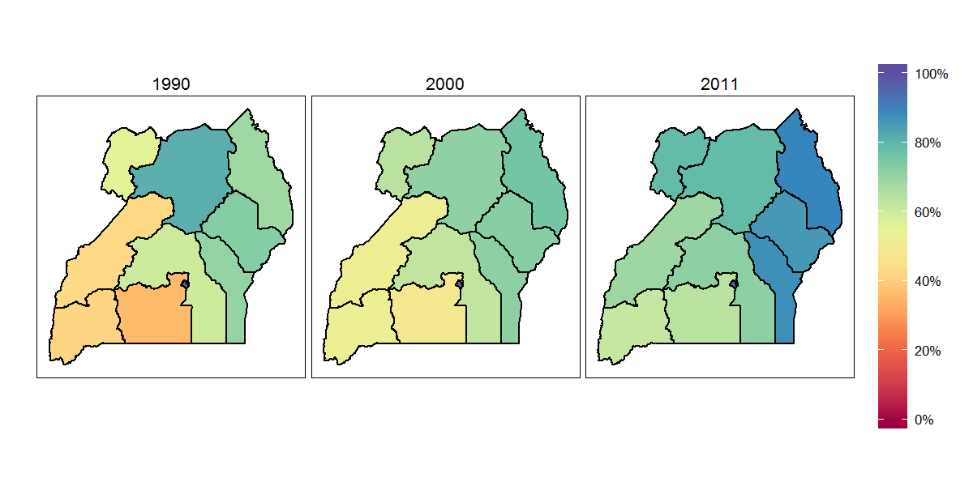
**

**Figure S3: Trends for indicators, 1990, 2000, and 2011**

**Under-5 mortality (deaths per 1,000 live births)**

**
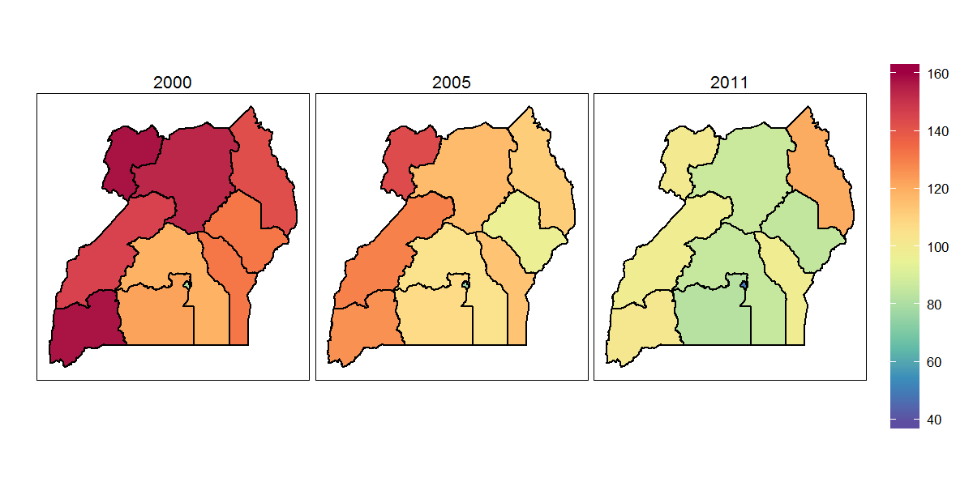
**

**Antenatal care (1 visit)**

**
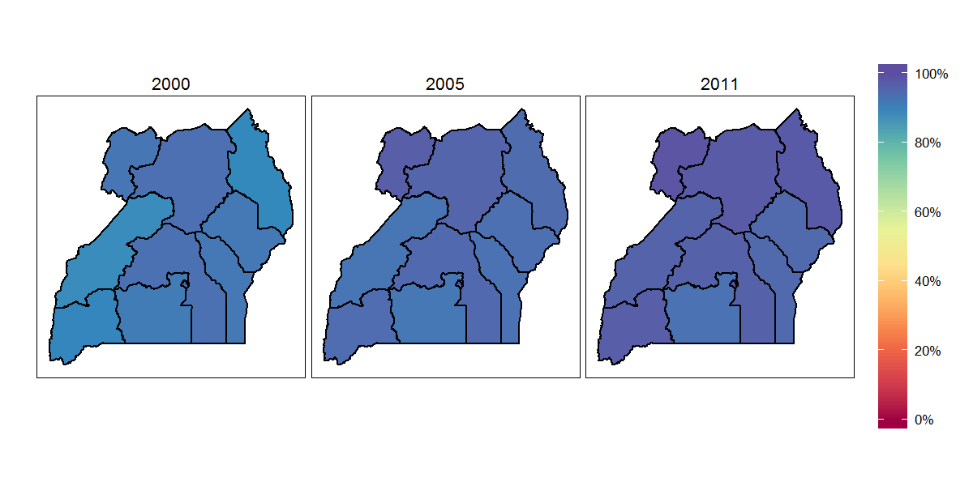
**

**Antenatal care (4 visits)**

**
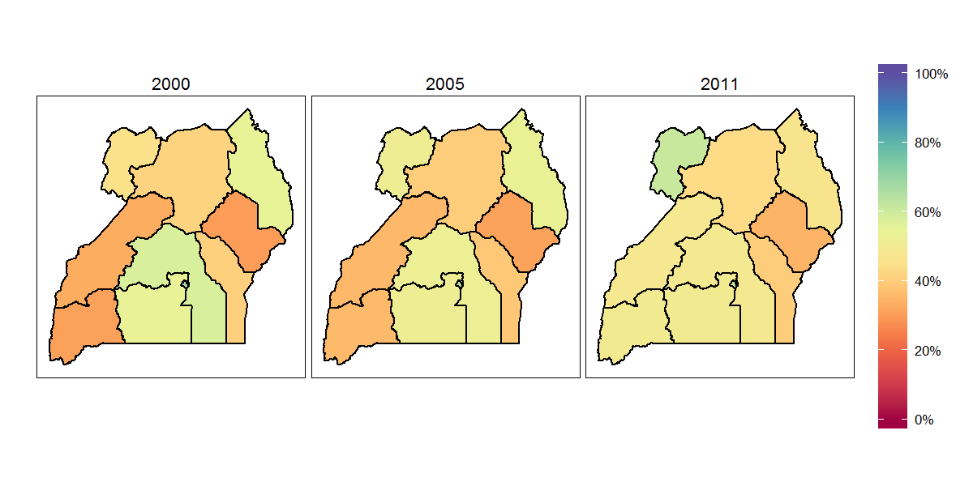
**

**Skilled birth attendance**

**
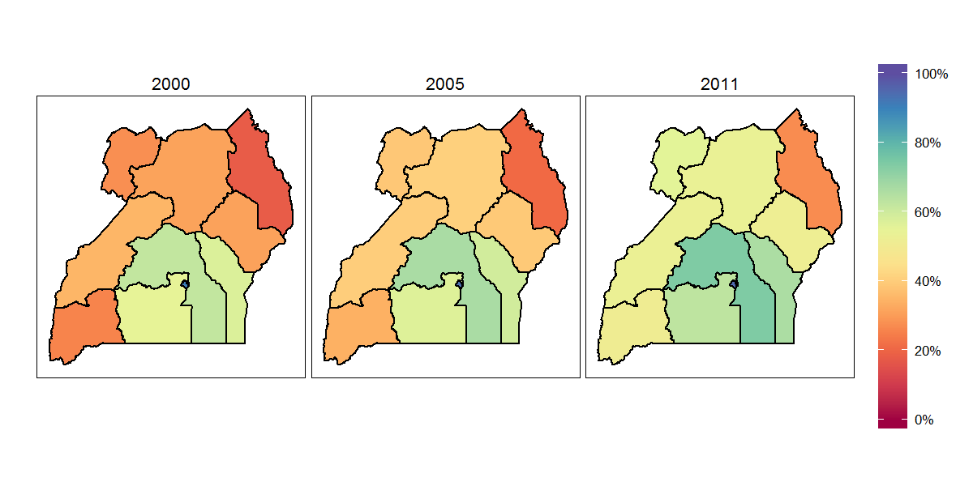
**

**BCG immunization (1 dose), children aged 0 to 12 months**

**
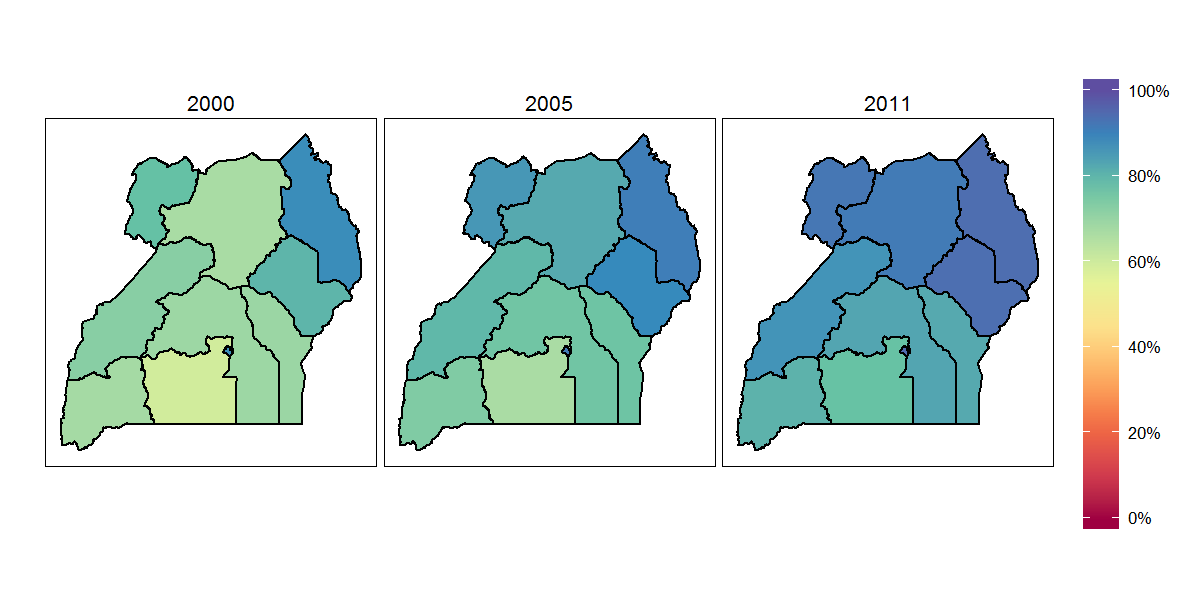
**

**BCG immunization (1 dose), children aged 0 to 59 months**

**
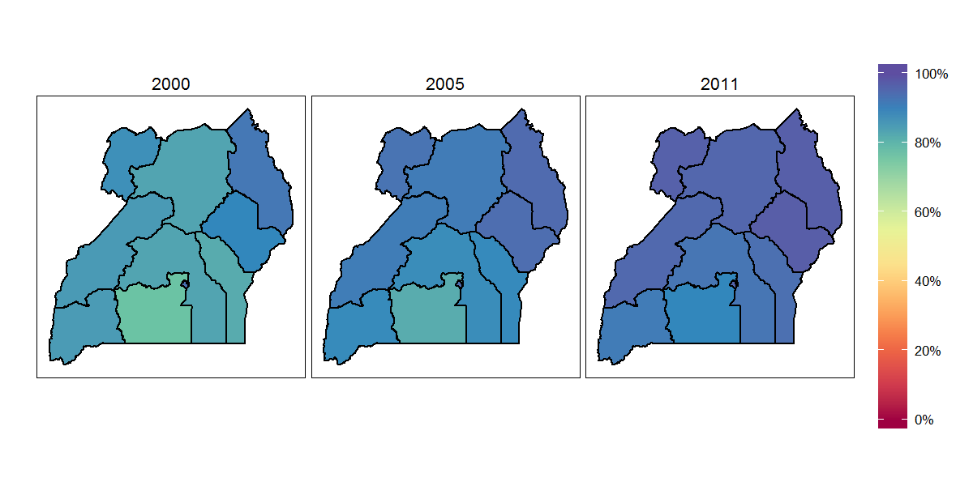
**

**Measles immunization (1 dose)**

**
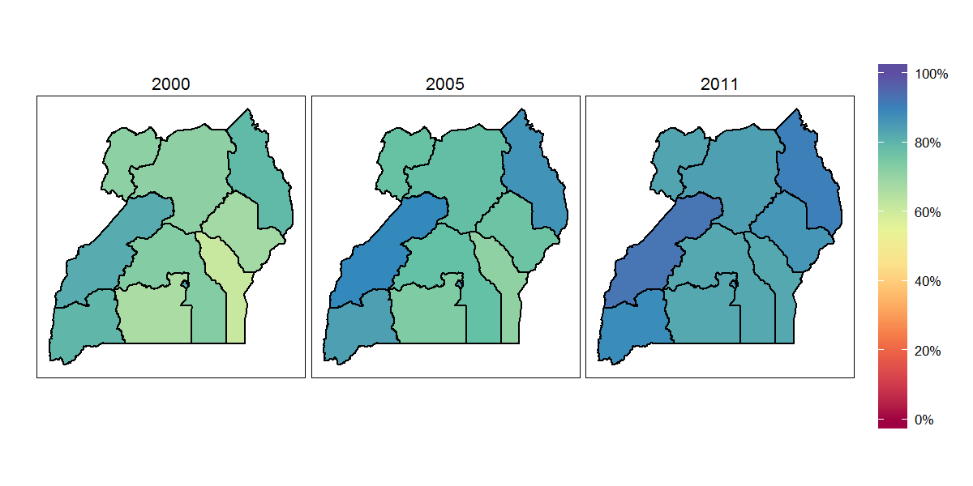
**

**Polio immunization (3 doses)**

**
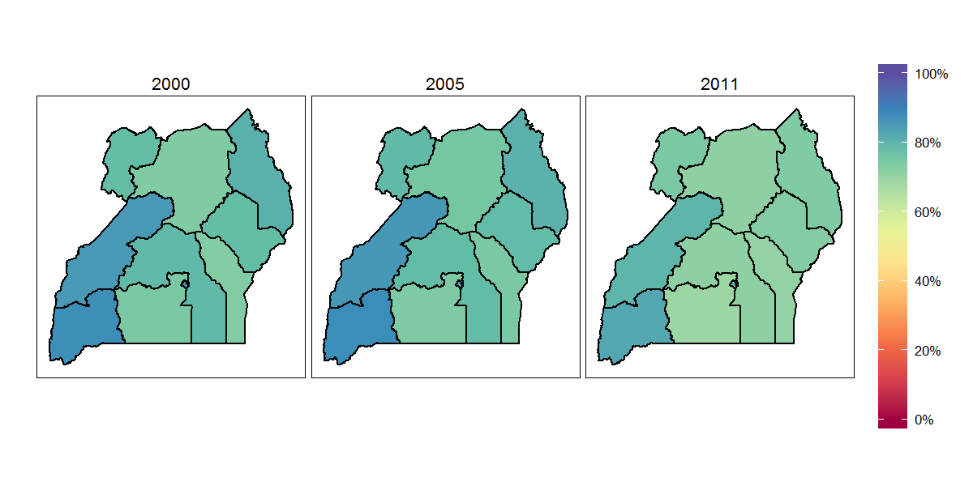
**

**Pentavalent immunization (3 doses)**

**
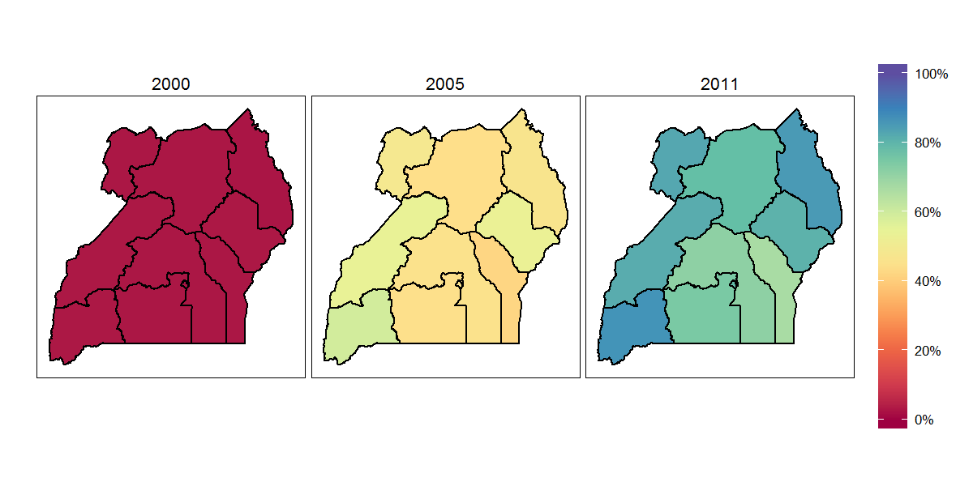
**

**Exclusive breastfeeding**

**
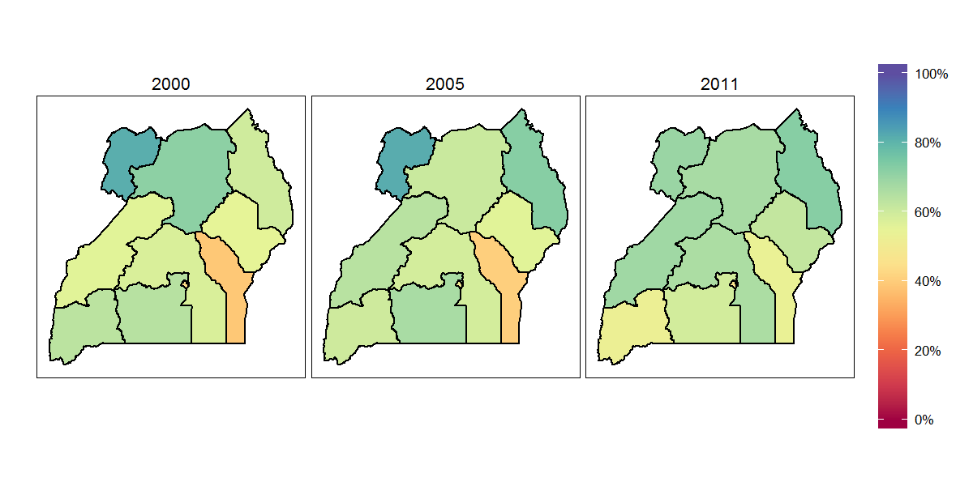
**

**Maternal immunization against tetanus (2 doses)**

**
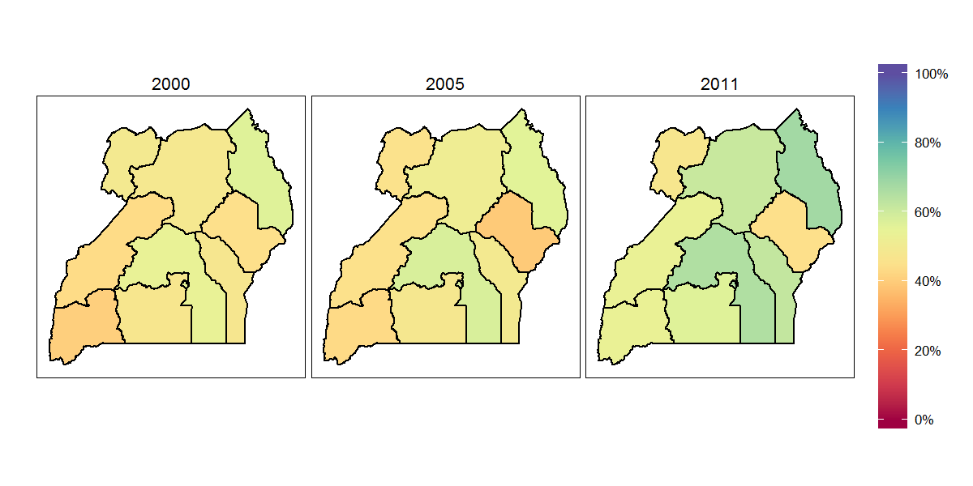
**

**Oral rehydration salts after diarrhea**

**
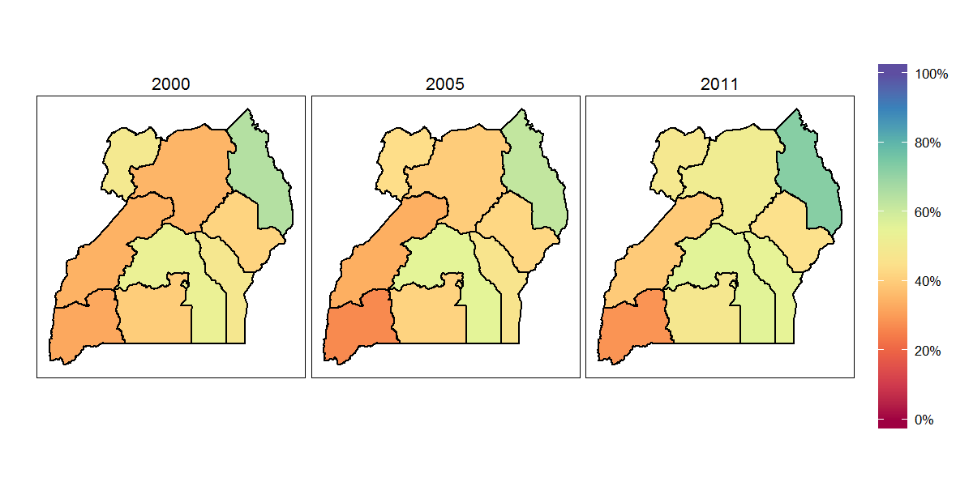
**

**Care at health facility after suspected acute respiratory infection**

**
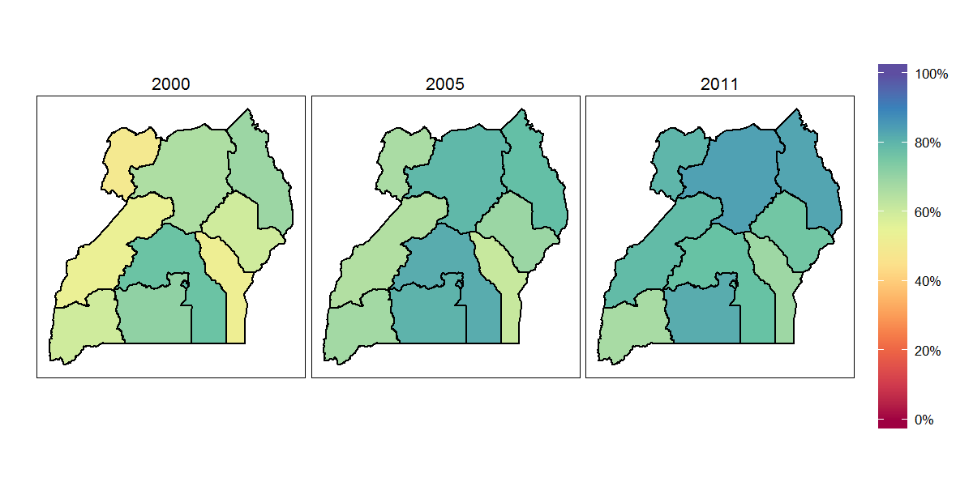
**

**Percentage of children underweight**

**
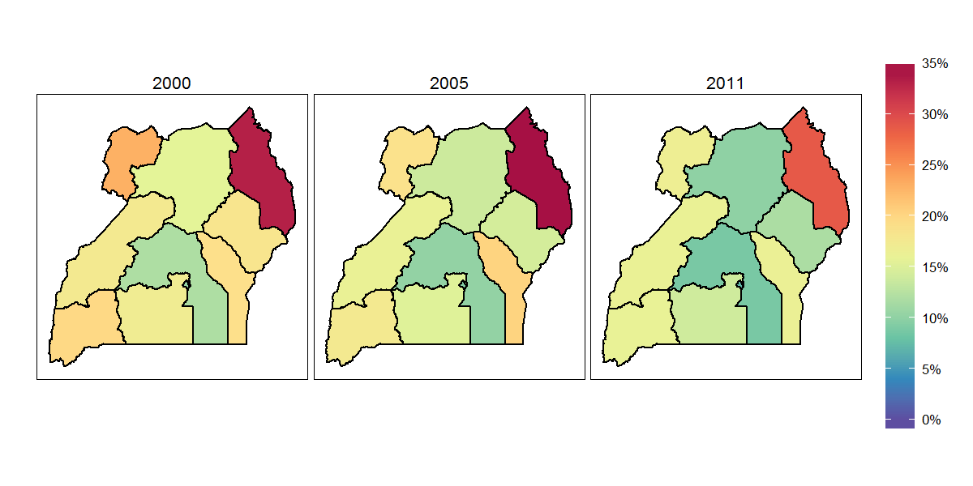
**

**Percentage of children stunted**

**
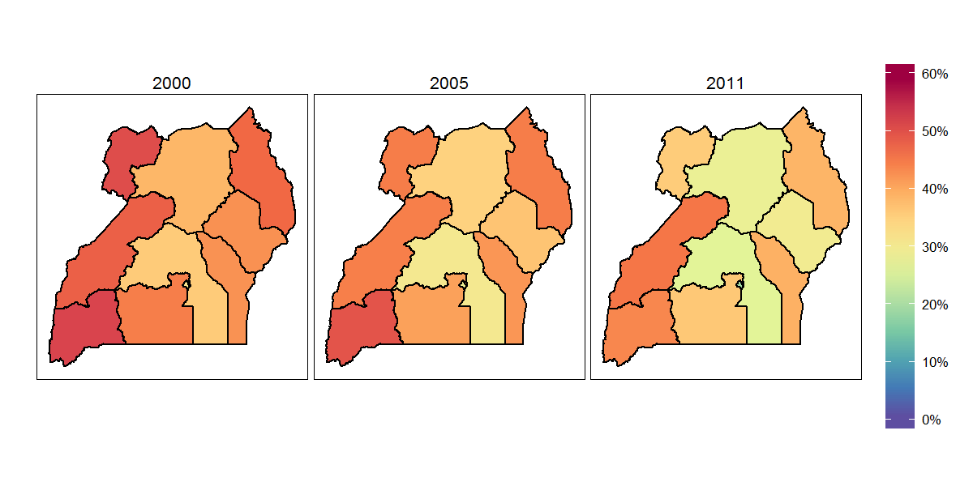
**

**Intermittent preventive therapy for malaria during pregnancy (1 dose)**

**
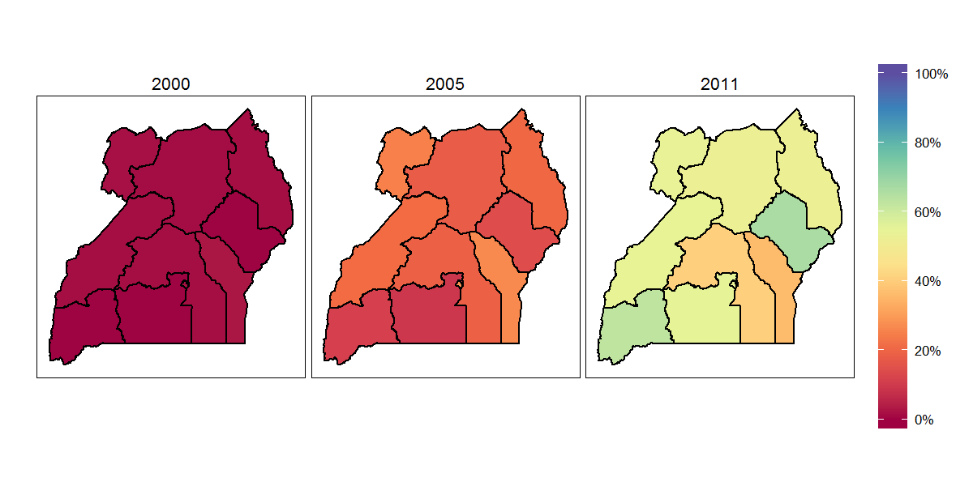
**

**Intermittent preventive therapy for malaria during pregnancy (2 doses)**

**
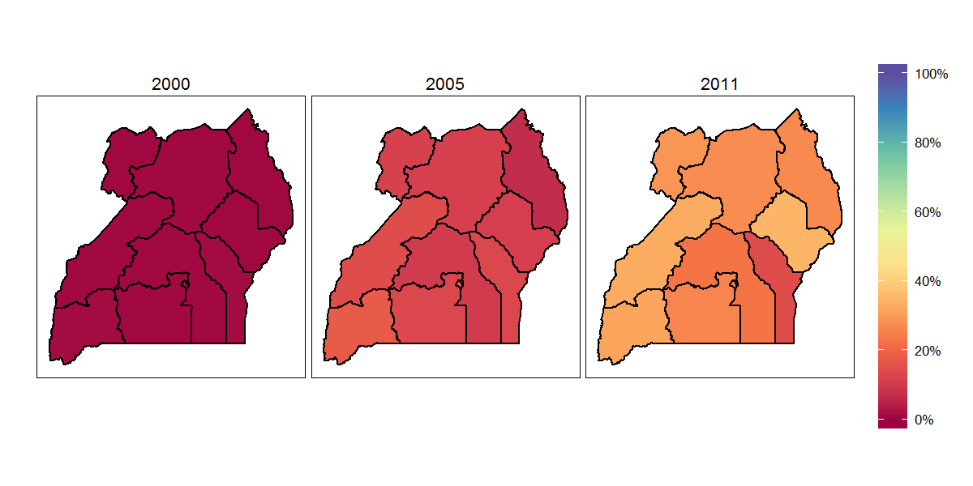
**

**Insecticide-treated net ownership**

**
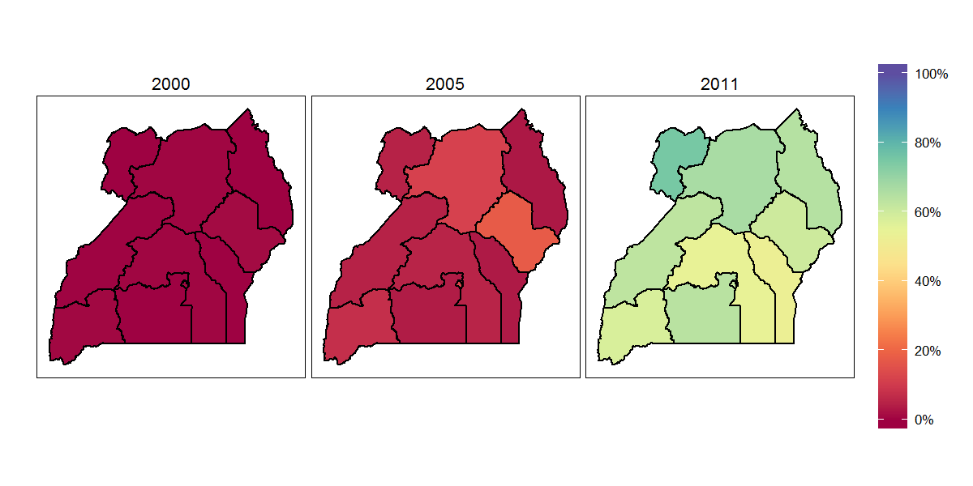
**

**Insecticide treated net use by children under 5**

**
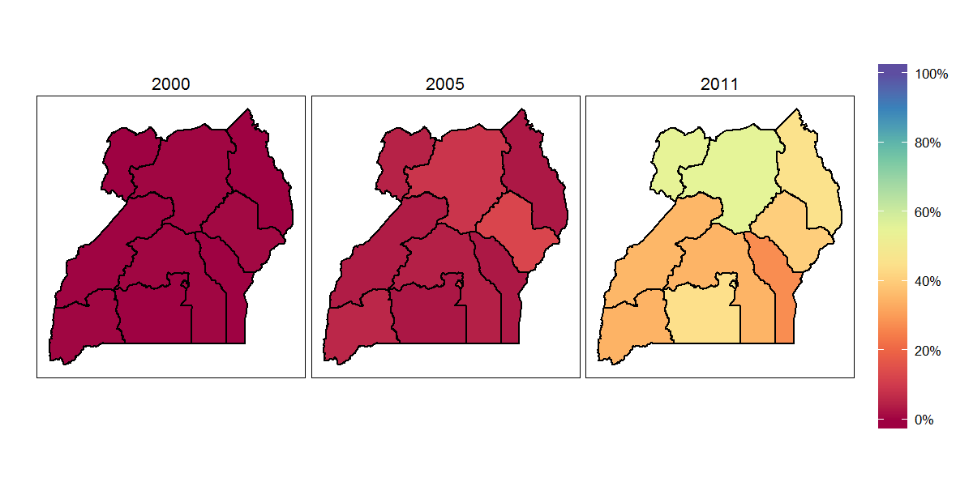
**

**Indoor residual spraying**

**
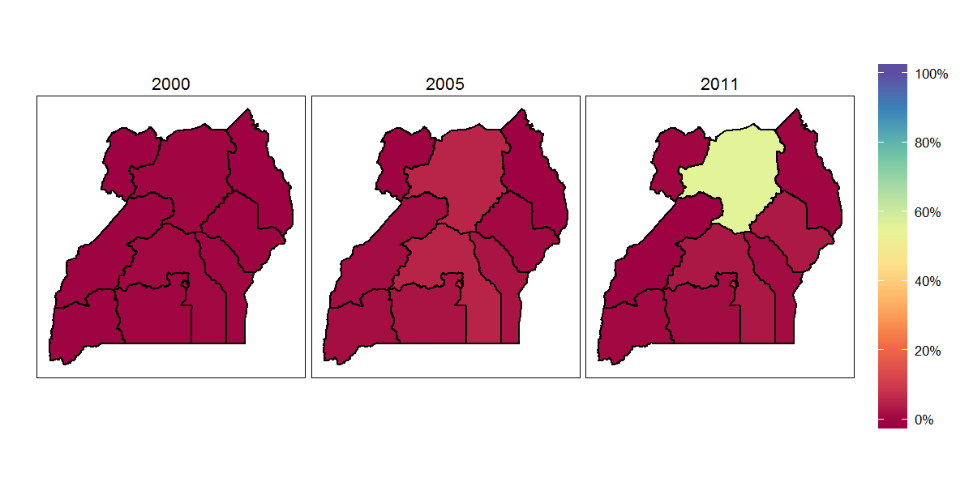
**

**Insecticide treated net ownership or indoor residual spraying**

**
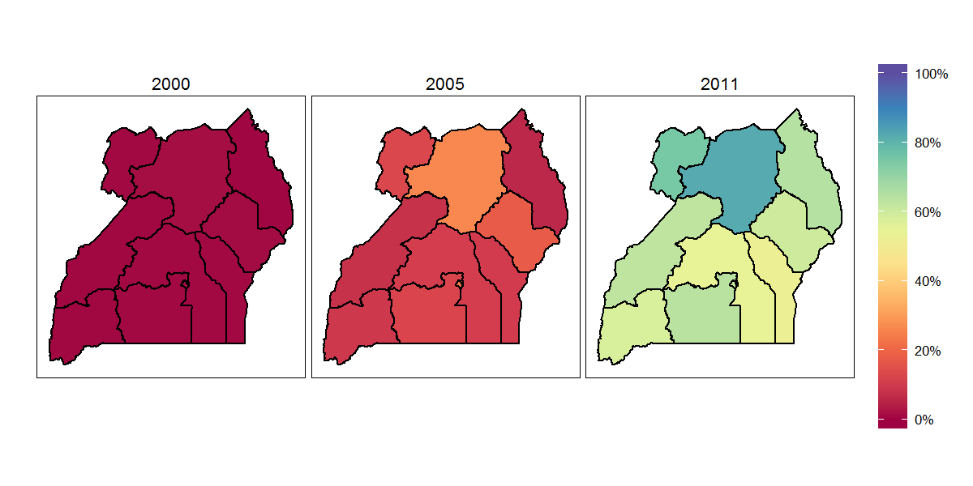
**

**Insecticide-treated net use by children under 5 or indoor residual spraying**

**
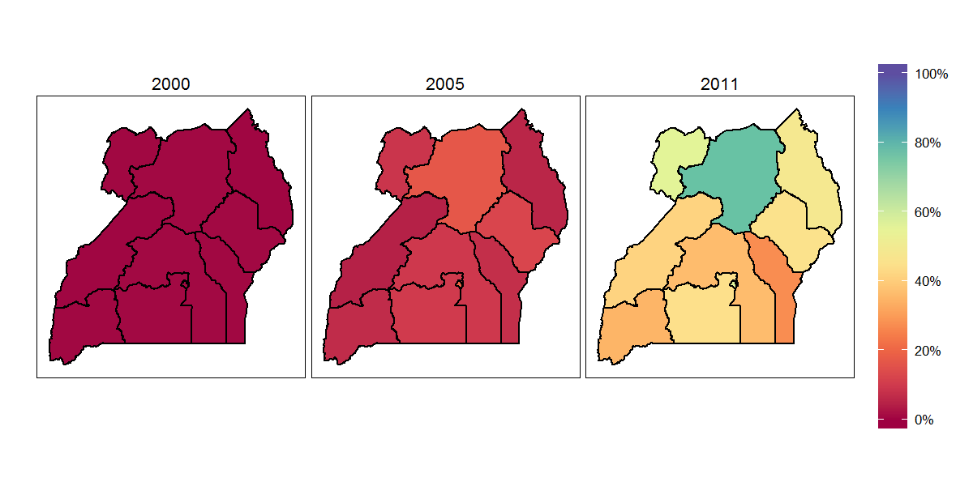
**

**Receipt of ACTs for febrile children under 5**

**
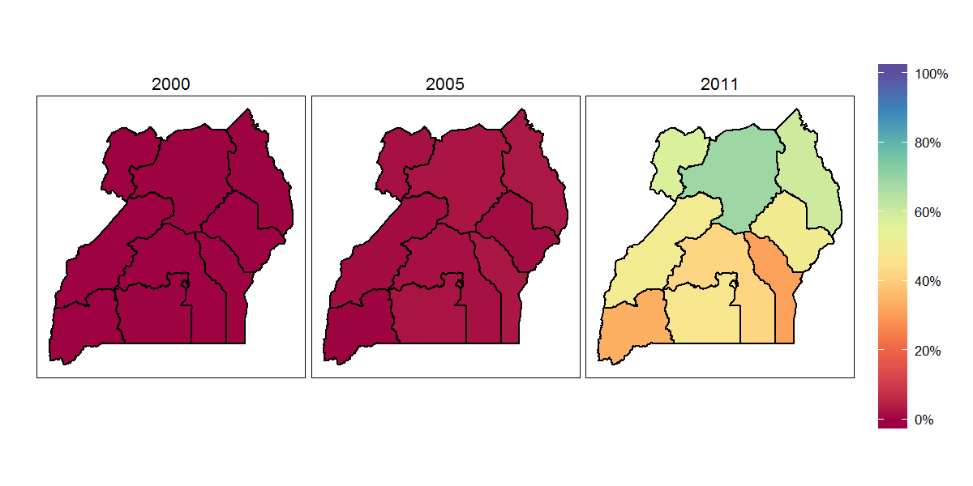
**

**Percent of antimalarials given to febrile children under 5 that were ACTs**

**
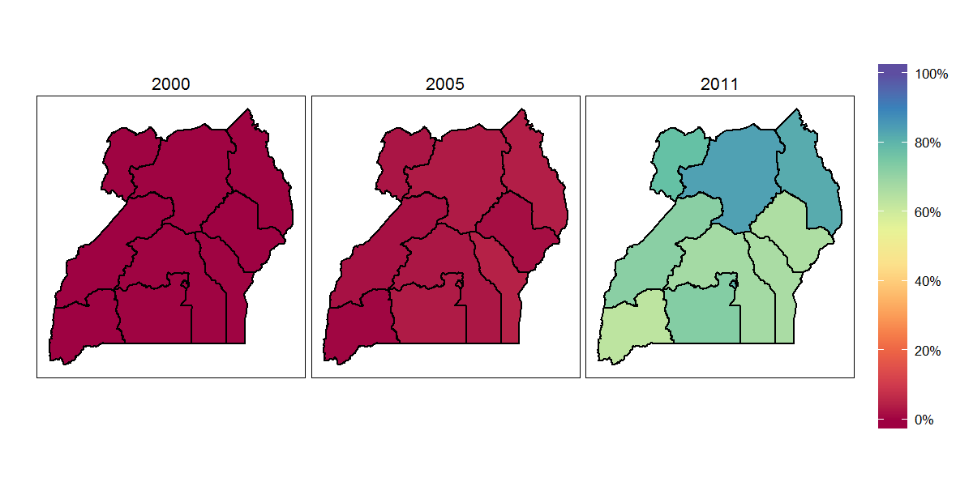
**

**Percentage of households with electricity**

**
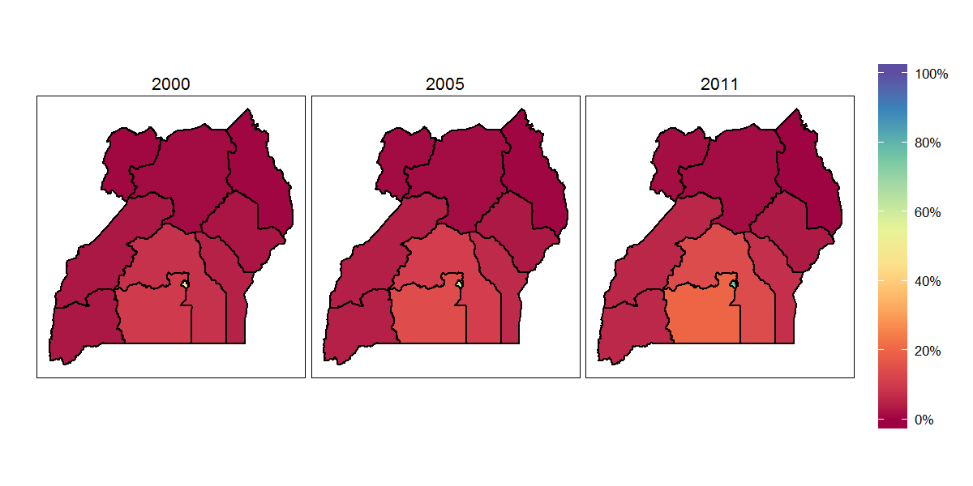
**

**Female headship of households**

**
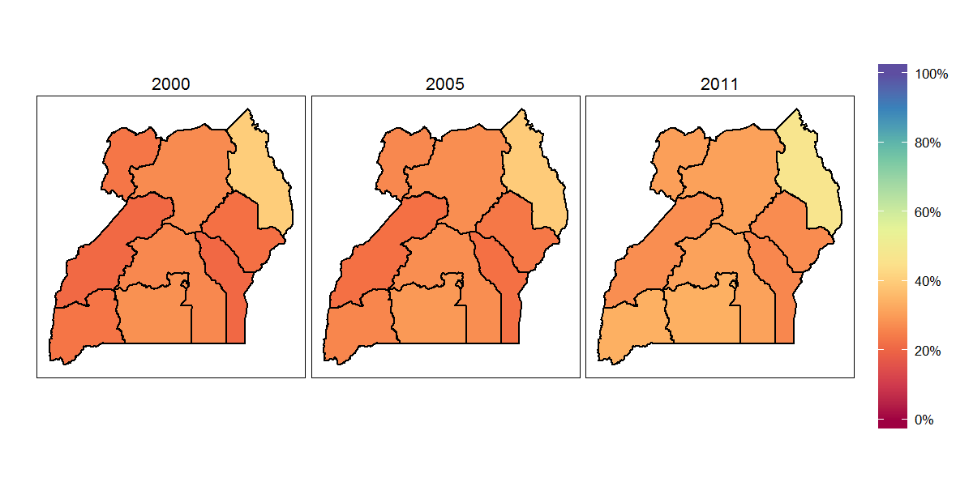
**

**Household size**

**
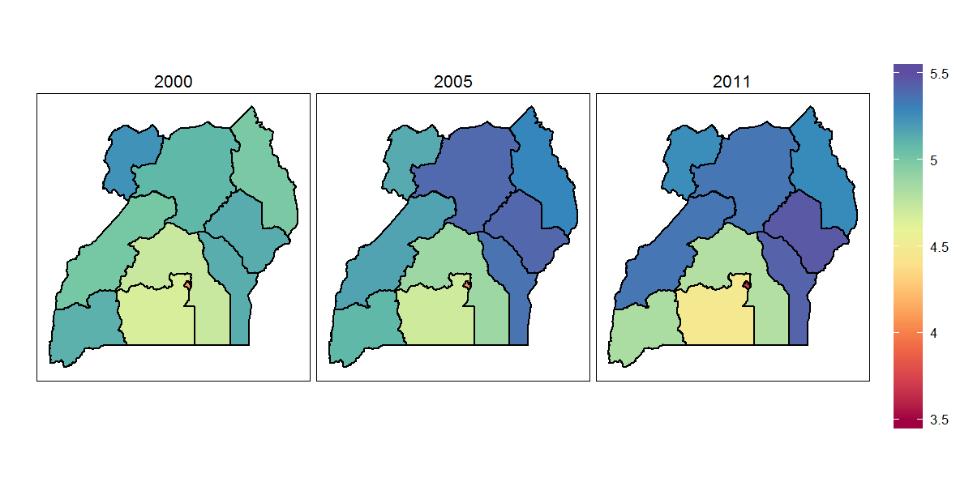
**

**Years of education of women 15 to 44 years old**

**
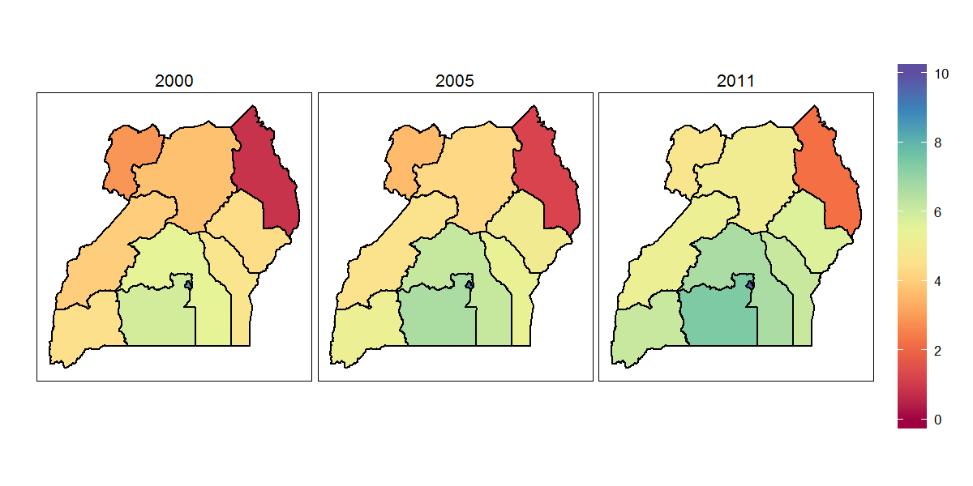
**

**Percentage of households with improved dwelling wall type**

**
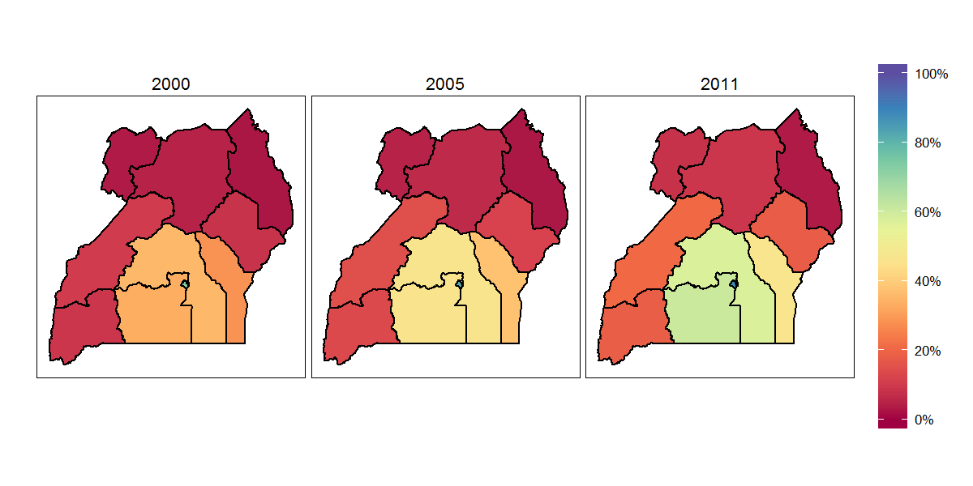
**

**Percentage of households with improved sanitation**

**
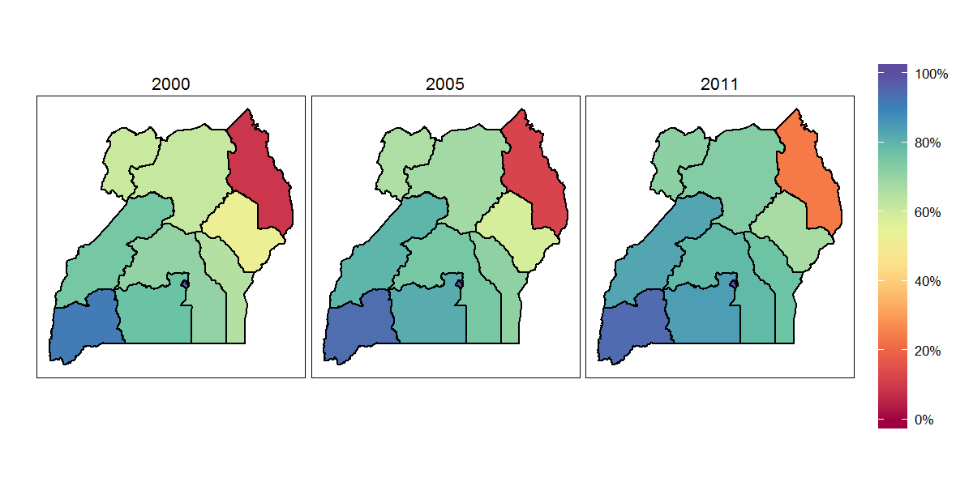
**

**Percentage of households with an improved water source**

**
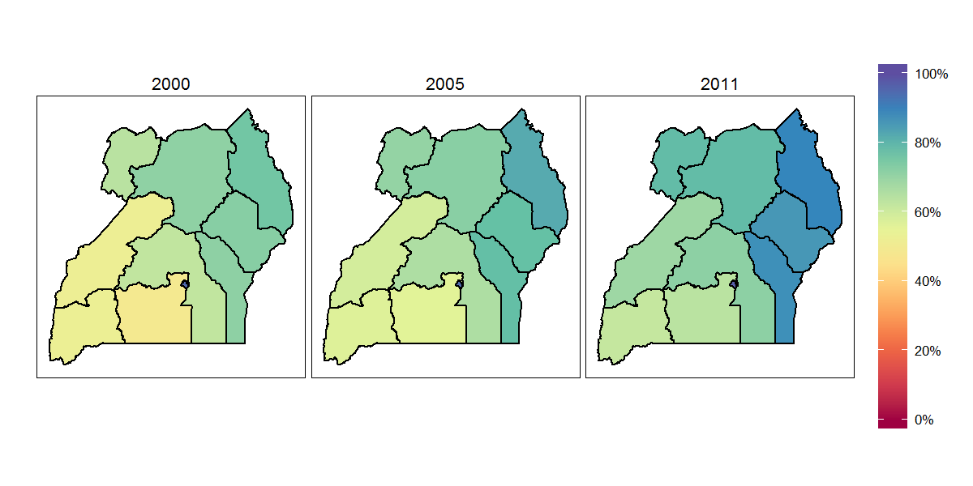
**
