# Supplementary figures and images for: Benchmarking health system performance across regions in Uganda: a systematic analysis of levels and trends in key maternal and child health interventions, 1990–2011
Source: BMC Med. 2015 Dec 3;13:285. doi: 10.1186/s12916-015-0518-x (PMC4668680; doi:10.1186/s12916-015-0518-x)

**Additional file 2**

**Figure S1: Demographic and Health Survey (DHS) 2011 region boundaries**


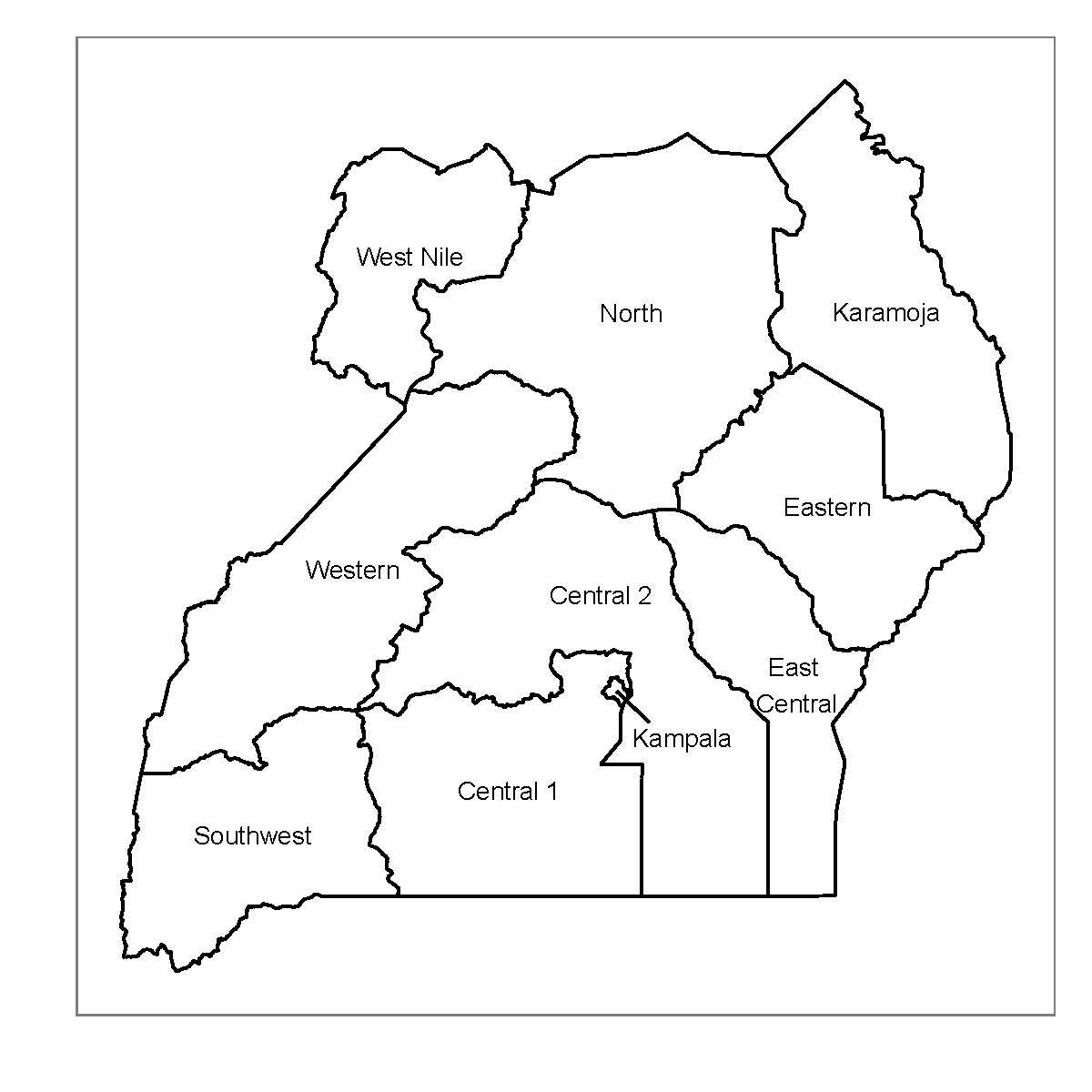

Supplement: Additional file 2: — Demographic and Health Survey (DHS) 2011 region boundaries. (DOCX 57 kb) [file 12916_2015_518_MOESM2_ESM.docx]
