# Supplementary material for: Benchmarking health system performance across regions in Uganda: a systematic analysis of levels and trends in key maternal and child health interventions, 1990–2011
Source: BMC Med. 2015 Dec 3;13:285. doi: 10.1186/s12916-015-0518-x (PMC4668680; doi:10.1186/s12916-015-0518-x)
Supplement: Additional file 3: — Comparing BCG immunization coverage across age groups by region. (DOCX 354 kb) [file 12916_2015_518_MOESM3_ESM.docx]

**Additional file 3**

**Comparing BCG immunization coverage across age groups by region**

Our analysis of BCG immunization included all children aged 0 to 59 months. This age group includes the largest sample size for estimating under-5 vaccination coverage. However, BCG vaccination is generally recommended at birth or within a child’s first six weeks of life. Subsequently, many indicators focus on the age group of 0 to 12 months, which is a stronger indicator for vaccination timeliness. To compare the effects of using different age groups, we estimated levels and trends of BCG coverage using age groups of 0 to 59 months and 0 to 12 months.

We found that BCG immunization coverage for children aged 0 to 12 months was consistently lower than for children aged 0 to 59 months (Figure S3), though the confidence intervals overlap for all regions. There are two plausible reasons for this difference. First, some children may be receiving BCG late, possibly through supplemental immunization campaigns. Previous studies in Uganda estimate that only about 9 in 10 children are vaccinated with BCG according to the country’s vaccination schedule [1, 2]. Second, it is also possible that maternal recall bias varies for children at different ages.

**Figure S3: BCG coverage for children aged 0 to 59 months (blue) and 0 to 12 months (red).**


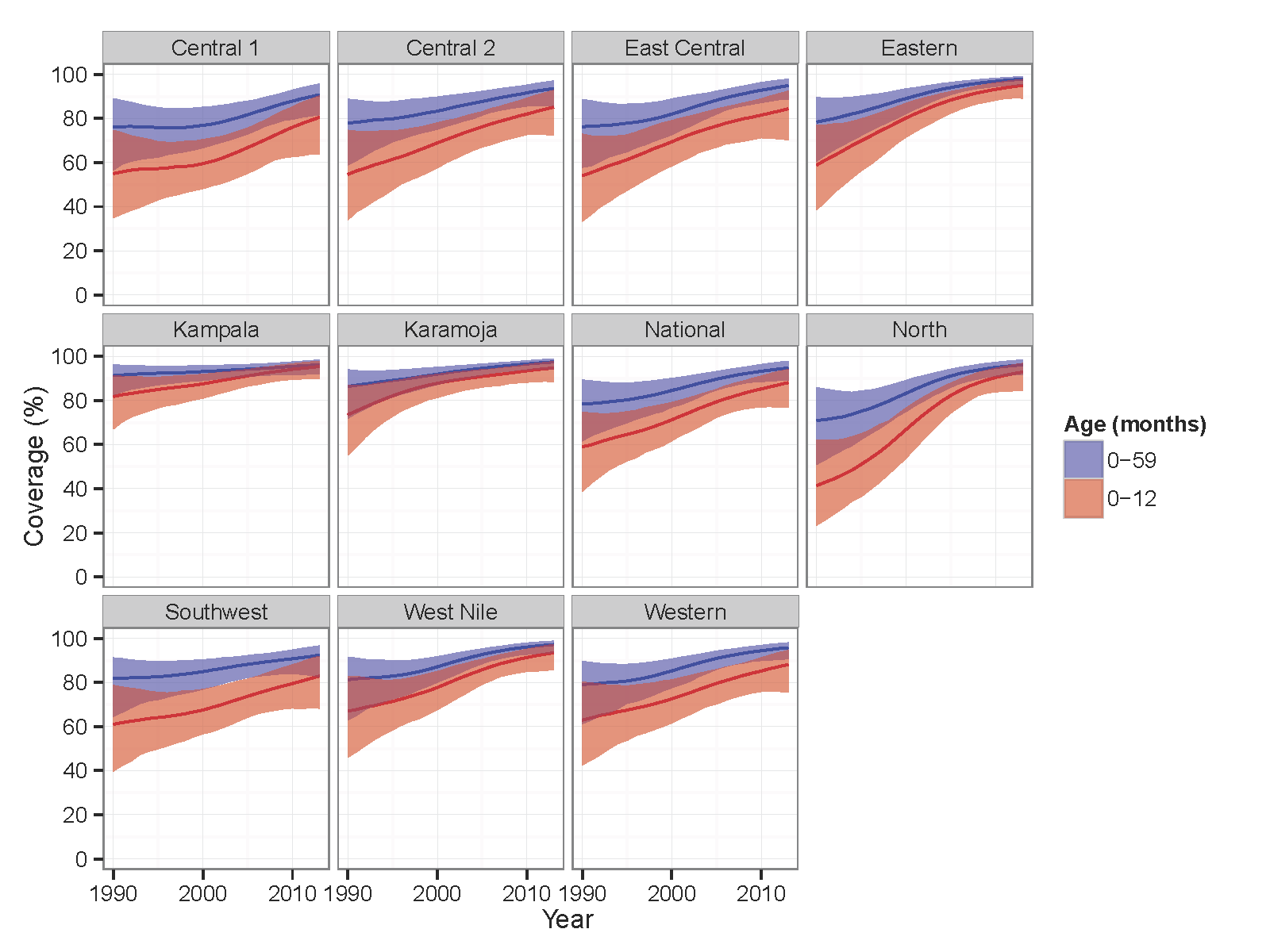


We found that the correlation coefficient for these two indicators, BCG coverage for children aged 0 to 12 months and 0 to 59 months, was 0.98, indicating that the measures have similar trends over time in all regions, with coverage in the 0-12 month age group consistently lower. In this study, we chose to use the 0 to 59 months age group for BCG immunization to best reflect overall coverage in children under 5. Results for both BCG indicators can be found in Additional file 1.

**References**

1. Fadnes LT, Nankabirwa V, Sommerfelt H, Tylleskär T, Tumwine JK, Engebretsen IMS: **Is vaccination coverage a good indicator of age-appropriate vaccination? A prospective study from Uganda**. *Vaccine* 2011, **29**:3564–3570.

2. Babirye JN, Engebretsen IMS, Makumbi F, Fadnes LT, Wamani H, Tylleskar T, Nuwaha F: **Timeliness of Childhood Vaccinations in Kampala Uganda: A Community-Based Cross-Sectional Study**. *PLoS ONE* 2012, **7**:e35432.
